# Genome streamlining via complete loss of introns has occurred multiple times in lichenized fungal mitochondria

**DOI:** 10.1002/ece3.5056

**Published:** 2019-03-21

**Authors:** Cloe S. Pogoda, Kyle G. Keepers, Arif Y. Nadiadi, Dustin W. Bailey, James C. Lendemer, Erin A. Tripp, Nolan C. Kane

**Affiliations:** ^1^ Department of Ecology and Evolutionary Biology University of Colorado Boulder Colorado; ^2^ Institute of Systematic Botany The New York Botanical Garden Bronx New York; ^3^ Museum of Natural History University of Colorado Boulder Colorado

**Keywords:** genome reduction, homing endonucleases, introns, lichen, parasitic genetic elements, symbiosis

## Abstract

Reductions in genome size and complexity are a hallmark of obligate symbioses. The mitochondrial genome displays clear examples of these reductions, with the ancestral alpha‐proteobacterial genome size and gene number having been reduced by orders of magnitude in most descendent modern mitochondrial genomes. Here, we examine patterns of mitochondrial evolution specifically looking at intron size, number, and position across 58 species from 21 genera of lichenized Ascomycete fungi, representing a broad range of fungal diversity and niches. Our results show that the *cox1*gene always contained the highest number of introns out of all the mitochondrial protein‐coding genes, that high intron sequence similarity (>90%) can be maintained between different genera, and that lichens have undergone at least two instances of complete, genome‐wide intron loss consistent with evidence for genome streamlining via loss of parasitic, noncoding DNA, in *Phlyctis boliviensis*and *Graphis lineola*. Notably, however, lichenized fungi have not only undergone intron loss but in some instances have expanded considerably in size due to intron proliferation (e.g., *Alectoria fallacina* and *Parmotrema neotropicum*), even between closely related sister species (e.g., *Cladonia*). These results shed light on the highly dynamic mitochondrial evolution that is occurring in lichens and suggest that these obligate symbiotic organisms are in some cases undergoing recent, broad‐scale genome streamlining via loss of protein‐coding genes as well as noncoding, parasitic DNA elements.

## 1 INTRODUCTION

Genome expansions and contractions are prominent, repeated occurrences across the tree of life, but the underlying mechanisms and selective regimes driving these changes are often unclear, limiting our ability to understand commonalities and differences across major domains (Adams & Palmer, 2003; Gray, Burger, & Lang, 1999; Jeffares, Mourier, & Penny, 2006; Khachane, Timmis, & Santos, 2007). Among the most prominent examples of variation in genome size and content is the mitochondrial genome (Bourque, Zdobnov, Bork, Pevzner, & Tesler, 2005; Ingman, Kaessmann, Pääbo, & Gyllensten, 2000; Palmer et al., 2000). The modern mitochondrial genome is derived from an ancient alpha‐proteobacterium, which, since its endosymbiosis with ancestral eukaryotes roughly 1.45 BYA (Martin & Mentel, 2010), has undergone significant reductions in genome complexity and size via loss of both protein‐coding genes and intronic sequences and intergenic spacers (Adams & Palmer, 2003; Gray et al., 1999; Khachane et al., 2007).

The extent of mitochondrial genome reduction varies substantially among taxa and can even vary between closely related sister species (Dibb, 1993; Jo & Choi, 2015; Lynch, Koskella, & Schaack, 2006; Signorovitch, Buss, & Dellaporta, 2007; Simmons et al., 2015; Wang, Zhang, Li, & Zhang, 2018). Bilateral metazoan mitochondrial genomes are highly consistent in size (16–20 kbp in length), usually contain the same 37 coding features, and lack introns or retrotransposable elements (Beagley, Okada, & Wolstenholme, 1996; Saccone, Giorgi, Gissi, Pesole, & Reyes, 1999). In contrast, other lineages of life, such as plants, have mitochondrial genomes that vary in content and size by up to three orders of magnitude (Alverson, Rice, Dickinson, Barry, & Palmer, 2011). Variations in content and size can be partially explained due to dynamic gains and losses of repetitive noncoding DNA (intergenic spacers) and selfish genetic elements (introns and transposable elements) that have parasitized portions of these genomes (Feschotte, Jiang, & Wessler, 2002; Paquin et al., 1997; Pogoda, Keepers, Lendemer, Kane, & Tripp, 2018). The differences in the presence/absence of these selfish genetic elements within the powerhouse organelle of eukaryotes are a major distinction between different broad evolutionary lineages.

There are two types of self‐splicing introns that are present in the mitochondrial genomes of most eukaryotic lineages, group I and group II, both of which are partial ribozymes and have the capability of moving themselves within the genome (Saldanha, Mohr, Belfort, & Lambowitz, 1993). In addition, both types of introns contain internal open reading frames (ORFs) that encode for intron‐encoded proteins (IEPs) that additionally help to promote the mobility of the introns that they occupy (Belfort, 2003; Belfort & Bonocora, 2014; Belfort, Derbyshire, Parker, Cousineau, & Lambowitz, 2002). Group I introns typically encode for homing endonucleases (HEGs) types LAGLIDADG and GIY‐YIG, while group II introns usually encode for reverse transcriptase genes (RT) (Lang, Laforest, & Burger, 2007). These genetic elements and other retrotransposable elements are often considered selfish as they pose no obvious value to their host genome (Edgell, Chalamcharla, & Belfort, 2011). However, because of their frequent replication and transposition throughout the genome, these genetic elements have the capability of introducing mutations within the host genome upon their insertion (Cambareri, Foss, Rowtree, Selker, & Kinsey, 1996; Nagy & Chandler, 2004). As such, these genetic elements have developed strategies that minimize mutation during insertion by avoiding initial disruption of the host exon–intron structure (Edgell et al., 2011). The HEG element can then function to spread both itself and its host intron throughout the genome (Burt & Koufopanou, 2004; Thiéry, Börstler, Ineichen, & Redecker, 2010) unless it is lost because of mutational events or host repression mechanisms (Brookfield, 2005; Chevalier & Stoddard, 2001). In addition, these elements are known to be able to move horizontally (Goddard & Burt, 1999; Wu & Hau, 2014) between different species genomes which helps to maintain their persistence.

Intron presence is well established in fungal mitochondrial genomes, but can vary widely among taxa (Giroux et al., 1994; Guha, Wai, Mullineux, & Hausner, 2017; Jeffares et al., 2006; Logsdon, 1998). Variation in intron number, which can occur even between different populations or strains of the same species, has the potential for widespread implications including impacting genome size and gene regulation or expression through alternative splicing mechanisms (Dibb, 1993; Jo & Choi, 2015; Lynch et al., 2006; Simmons et al., 2015; Wang et al., 2018). Among fungi, species can vary remarkably in intron content as well as genome size (Hensgens, Bonen, Haan, Horst, & Grivell, 1983; van der Veen et al., 1986; Fink, 1987; Derr, Strathern, & Garfinkel, 1991; Nielsen, Friedman, Birren, Burge, & Galagan, 2004; Guha et al., 2017; Wang et al., 2018; e.g., 18.9 kbp in *Schizosaccharomyces pombe*; Anziano, Perlman, Lang, & Wolf, 1983 and 235 kbp in *Rhizoctonia solani*; Losada et al., 2014). However, the study of intron evolution in fungi has been limited primarily to nonlichenized systems (Derr et al., 1991; Fink, 1987; Guha et al., 2017; Hensgens et al., 1983; Nielsen et al., 2004; van der Veen et al., 1986), despite the fact that tens of thousands of species of fungi are lichenized and have symbiotic lifestyles (Hawksworth & Hill, 1984).

The dynamics of gene gain and loss are sometimes amplified in organisms with mutualistic lifestyles, likely as a function of streamlining content and/or eliminating potentially competitive redundancies (Khachane et al., 2007; Pogoda et al., 2018; Senkler, Rugen, Eubel, Hegermann, & Braun, 2018; Tsaousis et al., 2008). Lichens are obligate symbiotic organisms that are geographically widely distributed, abundant, and ecologically important in most terrestrial ecosystems (Ahmadjian & Jacobs, 1981; Brodo, Sharnoff, & Sharnoff, 2001; Papazi, Kastanaki, Pirintsos, & Kotzabasis, 2015; Seaward, 1997). They consist of at minimum one primary mycobiont (typically an Ascomycete fungus) that provides structural protection for one or more primary photosynthetic partners (the photobiont: a green alga or cyanobacterium), which provide photosynthates to the mycobiont (Ahmadjian & Jacobs, 1981; Brodo et al., 2001; Papazi et al., 2015; Seaward, 1997). Present in most of Earth's terrestrial ecosystems (Papazi et al., 2015), the broad distribution and success of the lichen symbiosis contribute significantly to nutrient cycling and environmental bioindication (Fryday, Lendemer, & Howe, 2007; Kraichak et al., 2015; Nimis et al., 2018; Szczepaniak & Biziuk, 2003).

**Figure 1:**
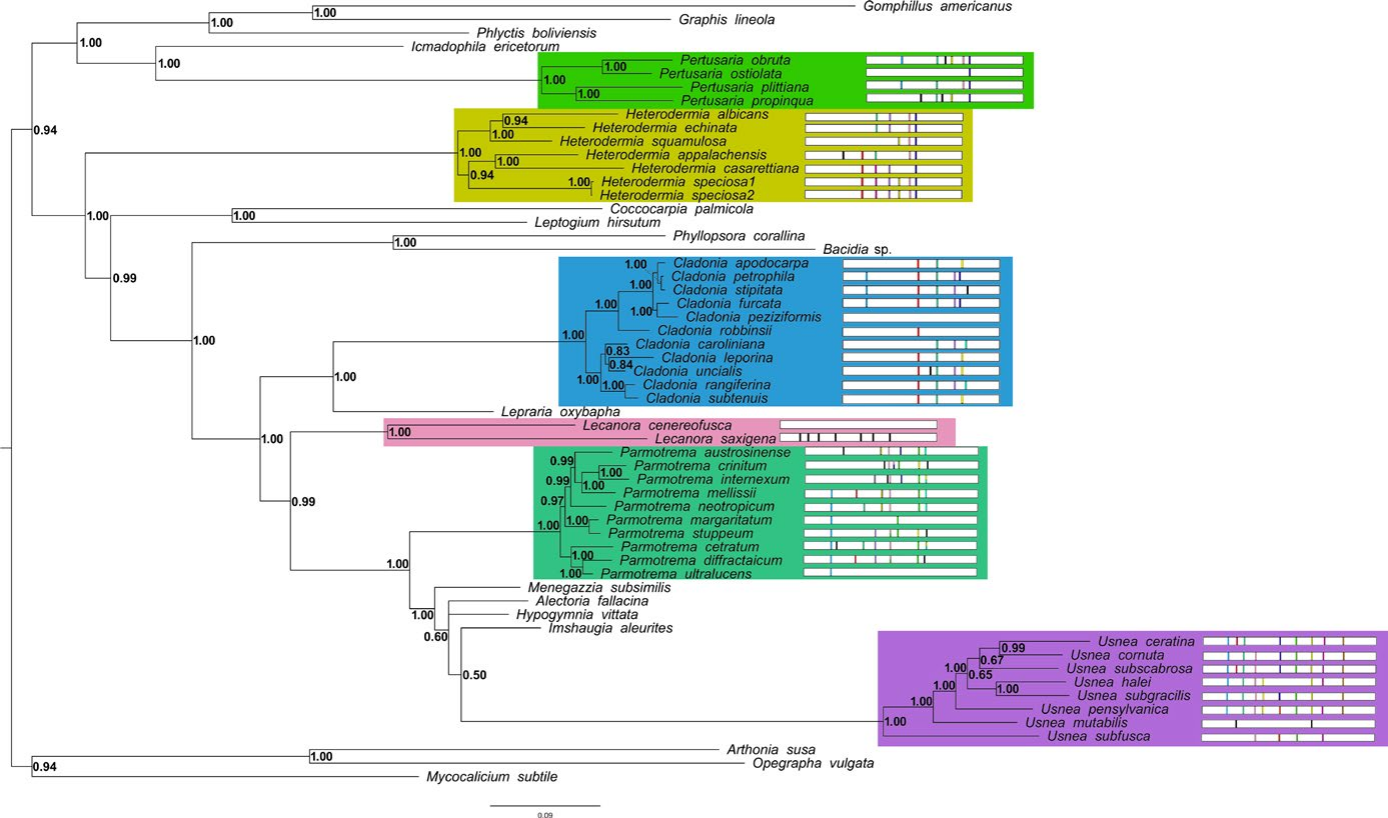
Fifty percent majority rule consensus tree from Bayesian analysis, with posterior probabilities mapped at each node. Tree rooted using *Artonia susa, A. ruana*, and *Opegrapha vulgata*.Genera for which multiple species were sampled are demarcated with colored boxes. To the right of each species is a cartoon representation of intron presence and location within the *cox1*gene. Sequence similarity between introns is represented by unique colors. A black‐colored intron indicates a unique, likely derived intron for that species. Introns are colored to indicate sequence similarity within a single genus (i.e., blue in one genus is not the same intron as blue in other genera)

Prior work characterizing mitochondrial evolution in lichens is limited but has revealed a highly variable landscape of introns across mycobionts (Brigham et al., 2018; Funk et al., 2018; Pogoda et al., 2018). Here, we employ data from 58 lichen mycobionts to examine broad‐scale patterns of intron gains, losses, and genome streamlining in seven different lineages of lichens: Lecanorales, Peltigerales, Telochistales, Ostropales, Pertusariales, Mycocaliciales, and Arthoniales (Figure 1). Specifically, we (a) record genome‐wide intron presence and sequence similarity in an evolutionary framework by inferring gains and losses through ancestral state reconstructions; (b) test the number of times complete or partial intron loss has occurred across the evolutionary history of the studied taxa; (c) examine intron sequence similarity and position in the *cox1*gene; and (d) quantify instances of genome streamlining via loss of selfish parasitic genetic elements, such as introns and homing endonucleases.

## 2 METHODS

### 2.1 Sample collection

To analyze intron gain and loss across lichenized fungal (i.e., mycobiont) mitochondrial genomes, we selected 58 Ascomycete species that span seven lineages of Lecanoromycetes. These 58 species represent 21 different genera. Twenty‐two mitochondrial genomes were previously sequenced and annotated (Pogoda et al., 2018); the remaining 36 genomes were newly assembled for the present study, and all are available on GenBank (Supporting Information Table S1). This taxonomically diverse dataset spans all major lichen morphologies (crustose, fruticose, and foliose growth forms), ecologies (growing on tree, rock, soil), and reproductive modes (sexual and asexual lineages).

All 58 species are native to the southern Appalachian Mountain biodiversity hotspot of eastern US area and were collected in the wild during fieldwork between 2016 and 2017. All specimens are deposited in the herbaria of the New York Botanical Garden (NY) and University of Colorado, Boulder (COLO) (Supporting Information Table S1). Efforts were made to sample only single thallus for both macro‐ and microlichens; however, due to the physically small size of microlichens, more than one individual was sometimes included. For macrolichens, ca. 1 × 1 cm of tissue was removed, targeting the thallus margins and lobes. For microlichens, tissue was scraped from rock or tree substrates using a sterile razor blade. Tissue samples were air‐dried in a laminar flow hood for 24 hr and then frozen at −20°C until transport to the University of Colorado for DNA extraction and sequencing.

### 2.2 DNA extraction and sequencing

Dried samples were first pulverized using tungsten carbide bearings in a Qiagen 96‐well plate shaker. Genomic DNA (gDNA) was extracted from tissues using a Qiagen DNeasy 96 plant kit. The manufacturer's protocol was modified to include a 10 min of 65°C incubation step for the ground material in lysis buffer, as well as a 100% ethanol wash, before final drying of the membrane prior to elution, which has been shown to improve DNA concentration and purity (Pogoda et al., 2018). Extracted samples were stored at −20°C prior to library preparation.

Genomic libraries were prepared following standard protocols using Nextera® XT DNA library prep kits (Illumina®), with 1 ng input DNA. Samples were barcoded using unique dual index adapters Nextera® i5 and i7. Libraries were cleaned using solid‐phase reversible immobilization (SPRI) to remove fragment sizes <300 base pairs. Quality control (QC) for pooled samples was conducted to ensure appropriate sample concentration and fragment size using a Qubit 3.0 fluorometer and an Agilent 2,100 Bioanalyzer. Pools that passed QC were normalized to a loading concentration of 1.8–2.1 p.m. with 1% PhiX control v3 added (Illumina®). All wet laboratory work was performed in the Department of Ecology and Evolutionary Biology at the University of Colorado, Boulder. Sequencing was conducted at the University of Colorado BioFrontiers Institute Next‐Generation Sequencing Facility in Boulder, Colorado.

### 2.3 Mycobiont genome assembly

Raw demultiplexed sequences were trimmed to exclude adaptor sequences using Trimmomatic‐0.36 using the parameters “ILLUMINACLIP:NexteraPE‐PE.fa:2:20:10MINLEN:140 LEADING:20 TRAILING:20” (Bolger, Lohse, & Usadel, 2014), with the file “NexteraPE‐PE.fa” containing the standard set of Nextera adapters to be trimmed from reads. Resulting fastq files were de novo assembled using SPAdes version 3.9.0 with the following parameters: careful ‐k 35,55,85 (Bankevich et al., 2012). The resulting assemblies included genomic representatives of all taxa (e.g., primary mycobiont, secondary fungal partners such as endolichenic and surficial fungi, bacterial symbionts, and photobionts) present in the metacommunity at the time of tissue sampling. Depth of the assembly was roughly proportional to the amount of input DNA such that the primary fungal and photobiont partners have the highest coverage in contrast to other symbionts.

We conducted several steps to ensure the mitochondrial sequences presented in this study belonged to the desired mycobiont rather than the photobiont or any other symbiont (such as endolichenic fungi) present in the metacommunity at time of sampling. First, we used command‐line BLAST to a representative lichenized Ascomycete mitochondrion (*Usnea ceratina*: NCBI accession NC_035940) to identify candidate contigs as mitochondrial, and these contigs typically had coverage of about 10–20 times that of nuclear genome contigs. Second, these contigs were then web BLASTed to the NCBI nonredundant database. In every taxon examined, the longest and highest coverage contigs identified with the command‐line BLAST had very high % identity (>95%) web‐BLAST hits to the expected lichenized fungus at common barcoding loci. Third, contigs were circularized using the raw genomic reads and error‐corrected using SAMtools *tview* (Li et al., 2009), and *tview* was used to ensure that no contigs assembled as chimeras between the mycobiont mitochondrion and another mitochondrion present in the meta‐assembly. Chimeric junctions appear as abrupt changes in alignment depth and sharp cutoffs in read alignments; *tview* revealed no chimerism in the assemblies.

Annotations were conducted using DOGMA (Wyman, Jansen, & Boore, 2004) and then prepared for submission in Sequin 15.10 (Bethesda MD) using sequences from representative genomes to confirm gene boundaries (*Cladonia rangiferina*: accession KY460674, *Heterodermia speciosa*: accession KY328643, *Lecanora saxigena*: accession MH359409, *Parmotrema stuppeum*: accession KY362439, *Pertusaria ostiolata*: accession: KY346830, and *Usnea ceratina*: accession NC_035940). The 58 lichen mitochondrial genomes were assembled and annotated by undergraduate and graduate students enrolled in University of Colorado's 2016 and 2017 Genomics classes taught by N. Kane and then examined for accuracy by the first and second authors. Specifically, each genome assembly was manually examined for sequence errors, completeness, and circularization (GeSeq was additionally utilized to confirm the quality and correctness of each annotation, Tillich et al., 2017). Annotation correctness was assessed by comparison within and among genera for each gene in each species, following the steps outlined in detail by Pogoda et al. (2018).

### 2.4 Genomic content

To assess gene and intron content for each mycobiont mitochondrion, gene boundaries and intron boundaries were identified using BLAST to determine exon/intron boundaries. The *cox1*gene was focused on in the analyses because it contained the greatest number of introns of any gene within each genome. Gene length, intron length, and sequence with homology to homing endonucleases (LAGLIDADG and GIY‐YIG) for the *cox1*gene were summed to determine overall length. For example, if there were eight ORFs with homology to a HEG (either full length or degenerated), these were summed to yield a total number of base pairs for that feature in each genome.

### 2.5 Genome correlations

In order to examine the drivers of genome size variation, we tested for correlation between genome size and (a) the summed *cox1*gene length, (b) the summed *cox1*intron length, (c) total number of introns in the *cox1*gene*,* (d) total number of introns present throughout the genome, (e) number of HEG elements present in the *cox1*gene, and (f) total number of base pairs of HEG elements in the *cox1*gene. Each test was conducted before and after correcting for phylogenetic relatedness using a phylogenetic generalized least squares (PGLS) approach under a Brownian motion model of trait evolution. PGLS tests were conducted using the R packages *ade4* (Dray & Dufour, 2007)*, ap* *e*(Paradis, Claude, & Strimmer, 2004)*, nlme* (Pinheiro, Bates, DebRoy, & Sarkar, 2017), and *geiger*(Harmon, Weir, Brock, Glor, & Challenger, 2008). To explore whether there exists a signal of evolutionary relatedness in each of our datasets relating to key genome features (Felsenstein, 1981), we tested for phylogenetic signal using Pagel's lambda and Blomberg's K (Blomberg, Garland, & Ives, 2003; Pagel, 1999). Analyses were conducted using the R package *phytools* (Revell, 2012), assuming a Brownian motion model of trait evolution.

### 2.6 Correlation between categorical data and intron number

To determine whether lichen growth form (macrolichen or microlichen; Supporting Information Table S1), photobiont partner (cyanobacterium, green coccoid alga, or green chain‐forming trentepohlioid alga; Supporting Information Table S1), or mode of reproduction (asexual or sexual; Supporting Information Table S1) was correlated with genome‐wide intron number and/or number of *cox1* introns, we conducted a one‐factor ANOVA test using the R package dplyr (Wickham, Francois, Henry, & Müller, 2016). Data were square root‐transformed prior to analysis to adjust for non‐normality of initial values. Character states were assigned to each species as follows: (a) All foliose and fruticose lichens were classified as macrolichens, and crustose lichens were classified as microlichens; (b) photobiont partners were assigned based on the primary photobiont present based on examination of the voucher specimen by JL and ET (note that no known tripartite lichens were included in this study); (c) reproductive mode was assigned based on the dominant reproductive mode present in both the specimen and the species (i.e., thalli and species that produced lichenized diaspores were assumed to reproduce asexually, even rare individuals in nature may also produce sexual reproductive structures; thalli and species that did not produce lichenized diaspores were treated as sexually reproducing because sexual reproductive structures were nearly always present and these were inferred to produce sexual spores).

### 2.7 Phylogenetic comparative analyses

To reconstruct a phylogeny to enable downstream analyses on intron evolution, we utilized data from the complete rDNA contig. First, full‐length or near full‐length nuclear rDNA contigs, which included sequences representing *18S*, ITS1, *5.8S*, ITS2, and *26S*, were extracted from the 58 metagenomic assemblies by performing a BLASTn of the meta‐assemblies against a representative rDNA contig (*Cladonia rangiferina*: accession KY119381). Because prior studies have shown that six of our study genera for which multiple representatives were sampled (*Cladonia, Heterodermia, Lecanora, Parmotrema, Pertusaria,*and *Usnea*) form strongly supported, reciprocally monophyletic lineages (Mower, Stefanović, Young, & Palmer, 2004), and to minimize potential impacts of paralogous introns at shared sites across different genera, we first aligned only the coding sequences for all 58 species ( i.e., 18S, 5.8S, and 26S). Second, the hypervariable regions (i.e., introns, ITS1 and ITS2) were aligned separately within each of these six genera and appended to the end of the coding sequence alignment. Intronic and noncoding data from other lineages (those with only one species per genus) were thus not considered in our alignment. Base positions for which more than one taxon was missing data were excluded from the alignment prior to phylogenetic analysis. The alignments were then combined into a single, joint matrix which was aligned using MUSCLE (Edgar, 2004) and then manually adjusted to correct for machine errors. The GTR + Γ+I model of sequenced was applied to all phylogenetic analyses as a result of model selection using the Akaike information criterion (AIC) implemented in ModelFinder (Kalyaanamoorthy, Minh, Wong, Haeseler, & Jermiin, 2017). Bayesian topologies were inferred in MrBayes (Huelsenbeck & Ronquist, 2001; Ronquist & Huelsenbeck, 2003), sampling trees over 1,000,000 MCMC generations (Nei & Kumar, 2000) and treating gaps as missing data. The default first 25% of trees representing the burn‐in were excluded from further consideration. The sampling temperature was set to temp = 0.002, and eight chains were implemented in the tree search. The posterior distribution of trees was used to calculate a 50% majority rule consensus tree, upon which we mapped Bayesian posterior probabilities (Tamura & Nei, 1993). The tree was rooted using *Arthonia ruana, A. susa,* and *Opegrapha vulgata*[Class Arthoniomycetidae]. Final matrices used in our phylogenetic analyses are available on Zenodo (1,420,516).

### 2.8 Intron positions and sequence similarity within a genus

To assess how conserved introns were within and across species, intron positions within the *cox1*gene for each genus were mapped onto the resultant majority rule phylogenetic tree by conducting BLASTx searches of a representative sequence of *cox1* (*Cladonia rangiferina*: accession KY460674, *Heterodermia speciosa1*: accession KY328643, *Lecanora saxigena*: accession MH359409, *Parmotrema stuppeum*: accession KY362439, *Pertusaria ostiolata*: accession KY346830, and *Usnea ceratina*: accession NC_035940) against each species and recording the relative intron positions within the gene. These intron sequences were compared for nucleotide similarity using BLAST and then colored based upon intron similarity (i.e., the “red” intron in *Cladonia*has high sequence similarity only within that genus and is not the same intron as “red” in another genus (Figure 1).

### 2.9 Mycobiont intron search in metagenomic assemblies

To assess whether the introns that were present in the mitochondrial genomes of the mycobiont were present in other genomes (e.g., the mitochondrial genome of the photobiont or the nuclear mycobiont genome), a command‐line BLASTn was performed using a concatenated file containing all the sequences from the introns extracted from each of the mycobiont mitochondrial genomes against the meta‐assemblies of each of the 58 species. The resulting BLAST tables were parsed, and each hit was assessed for bit score. We determined the species from which the contig came by using BLASTn searches against the NCBI nonredundant database.

### 2.10 Intron clustering

**Figure 2:**
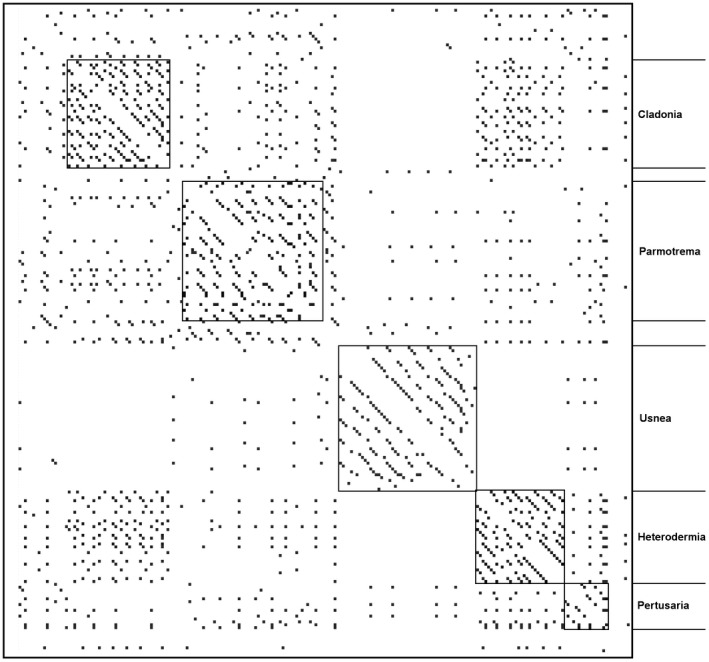
Pairwise similarity matrix of the resulting bit score from comparison between introns in the *cox1*gene of each species that was present within a genus here represented by two or more species (rows and column each represent unique introns). Matrix represents a nucleotide all‐versus‐all BLASTn (diagonal values representing identical comparisons omitted). Gray scale is weighted by bit score (measure of sequence similarity and number of bp that are similar); darker colors indicate higher bit score. Within‐species comparisons are demarcated by boxes, and genus is noted on right‐hand side of figure.

Intronic DNA sequences for the *cox1*gene were extracted from each annotation to compare sequence similarity for the gene between all 58 species. An all‐versus‐all BLASTn was conducted, and the resulting table was parsed to include only hits >100 bp in length and with a bit score >100. A pairwise similarity matrix was generated in which the bit score of the comparison between two introns was used to produce grayscale weighting for the cell representing the comparison (i.e., black indicates higher sequence similarity than light gray; Figure 2).

**Figure 3:**
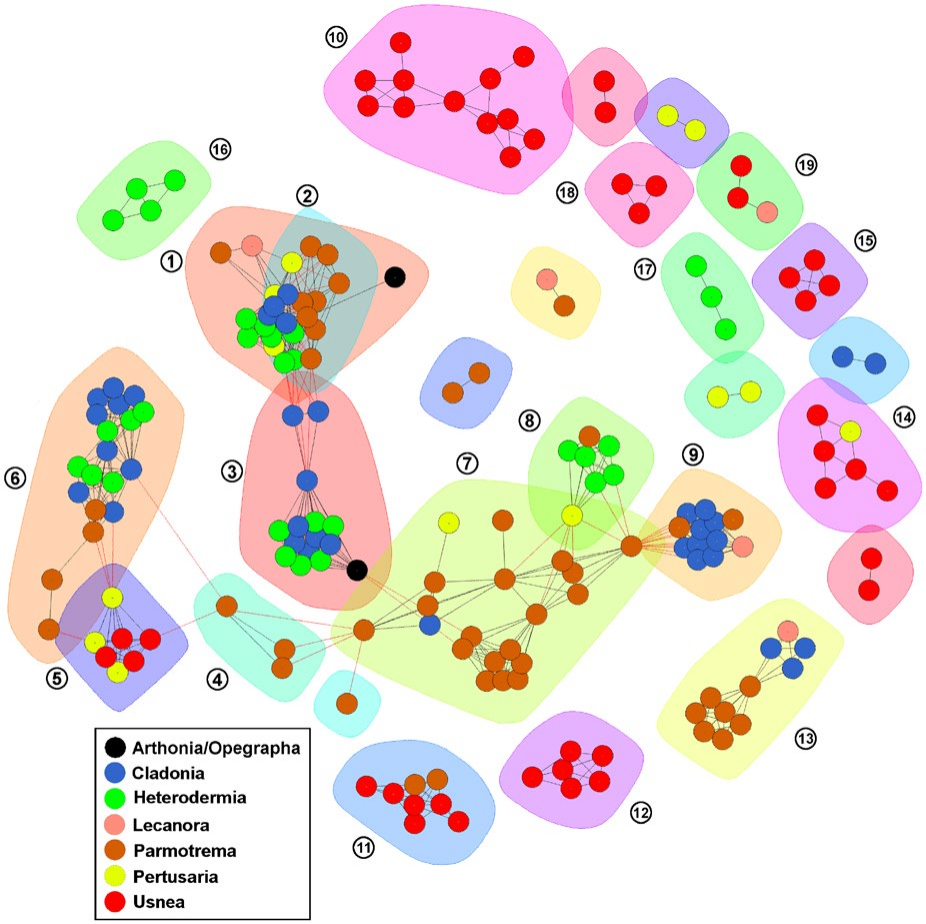
Clustering of *cox1*gene introns with high sequence similarity between species. Clusters with more than one genus (more than one color dot) indicate ancestral introns, while clusters with introns from only one genus (one color dot) are more recent gains. Clusters of greater than two introns are numbered, and vertices (nodes) are colored to represent the species that the intron originated from

Introns were clustered using the R program *iGraph* (Csardi & Nepusz, 2006). The function *cluster_optimal* was employed to calculate the optimal community structure for the intron sequences that resulted from the all‐versus‐all command‐line BLAST. A bipartite graph was constructed with vertices representing introns and edges between vertices representing BLAST similarity weighted by bit score. Each intron was color‐coded to identify the genus from which it originated (Figure 3).

### 2.11 Ancestral state reconstruction

**Figure 4:**
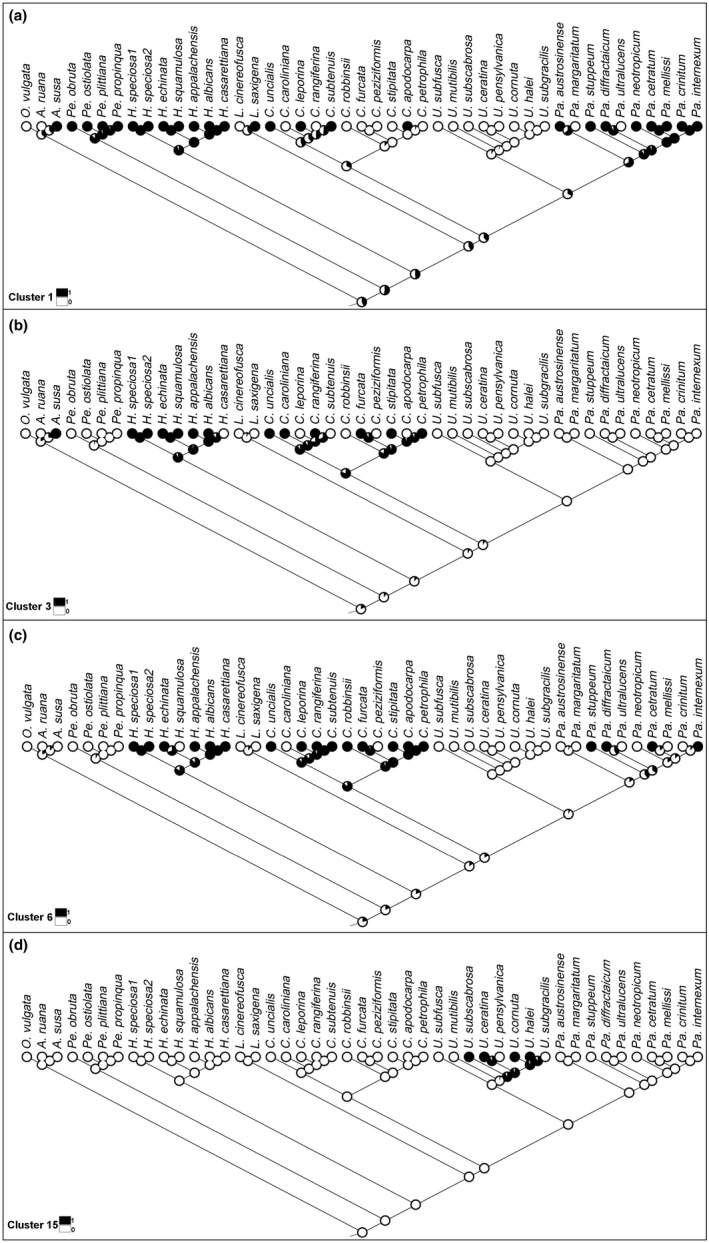
Ancestral state reconstruction for four of the nineteen intron clusters (these clusters were chosen to demonstrate early and late intron gains): clusters 1 (a), 3 (b), 6 (c), and 15 (d; see Figure 3 for cluster identification). Pies at nodes represent likelihoods that a given intron cluster was (black) or was not (white) present at ancestral node. Tree shows only species having introns with sequence homology to other species (see text for further explanation)

To assess the evolution of intron sequence similarity, as well as broad‐scale gain and loss events, ancestral state reconstructions (Ekman, Andersen, & Wedin, 2008) were conducted using Mesquite (Maddison & Maddison, 2018). The Bayesian consensus tree was imported and trimmed to only include species that contained *cox1*introns and had more than one representative per genus. A character matrix of the 19 *cox1*intron clusters (see Intron Clustering) was built for these 45 species; for each species, we scored whether the cluster was (1) present or (0) absent. The history of each character was reconstructed using maximum‐likelihood methods to estimate ancestral states, with default probability models in effect. Nodes (internal and external) were colored (black or white) to indicate the presence or absence of a given character (i.e., intron; Figure 4).

A character matrix for total intron length, total intron number in the *cox1*gene, and genome‐wide total intron number was imported to assess overall ancestral intron gain and loss. The steps outlined above were repeated to reconstruct the ancestral states of these characters. Nodes (internal and external) were color‐coded to indicate the range of *cox1* intron lengths (Supporting Information Figure S2a), *cox1*intron number (Supporting Information Figure S2b), and genome‐wide intron number (Supporting Information Figure S3).

## 3 RESULTS

### 3.1 Mycobiont genome content

Each of the 58 lichen mitochondrial genomes contained a conserved set of 14 protein‐coding genes: c*ob, cox1, cox2, cox3, nad1, nad2, nad3, nad4, nad4L, nad5, nad6, atp6, atp8*, and *rps3*. Another protein‐coding gene present in some but not all the genomes was *atp9,*which we showed previously to be absent in some members of Arthoniales (Bailey, D. W., Nadiadi, A. Y., Keepers, K. G., Pogoda, C. S., Lendemer, J. C., Kane, N. C., Tripp, E. A. ms in prep.), Lecanorales, Ostropales, and Teloschistales (Pogoda et al., 2018). Genome‐wide, the number of introns varied markedly in comparison with the number of genes, from no introns in *Phlyctis boliviensis*and *Graphis lineola* to 23 in *Parmotrema neotropicum*(Table 1). The total number of introns was correlated with overall genome size (*R* ^2^ = 0.49, *p* = 0.0001) and remained significant after correcting for phylogenetic relatedness (*p* = 0.0001). Eleven of the 15 genes were parasitized by introns, but four (*atp8, atp9, nad4L,*and *nad6*) did not contain any introns in the species examined.

**Table 1:** Species list, mitochondrial genome size (bp), number of introns in the cox1 gene, and the number of HEGs in the *cox1*gene. Quantification of number of introns in the other mitochondrial genes present, total number of genes, and total genome‐wide number of introns in each species

Species	Genome Size (bp)	Number of *cox1* Introns	Number of HEGs in *cox1*	*cox2*	*cox3*	*cob*	*atp6*	*atp8*	*atp9*	*nad1*	*nad2*	*nad3*	*nad4*	*nad4L*	*nad5*	*nad6*	*rps3*	Total Number of Genes	Total Number of Introns
*Alectoria fallacina*	75,417	7	6	1	1	1	1	0	0	2	0	0	0	0	2	0	7	14	22
*Arthonia ruana*	27,342	0	4	0	0	0	0	0	0	0	0	0	1	0	0	0	1	15	2
*Arthonia susa*	50,798	6	0	0	0	2	0	0	0	0	0	0	0	0	2	0	1	15	11
*Bacidia sp*.	38,546	1	0	0	0	0	0	0	X	1	0	0	0	0	0	0	1	14	3
*Cladonia apodocarpa*	50,456	3	3	0	0	0	0	0	0	0	0	0	0	0	0	0	2	15	5
*Cladonia caroliniana*	51,501	3	3	0	0	0	0	0	0	1	0	0	0	0	0	0	0	15	4
*Cladonia furcata*	59,877	5	3	0	0	3	0	0	0	0	0	0	0	0	1	0	2	15	11
*Cladonia leporina*	50,045	3	3	0	0	0	0	0	0	0	0	0	0	0	1	0	1	15	5
*Cladonia petrophila*	53,100	5	4	0	0	0	0	0	0	1	0	0	0	0	0	0	2	15	8
*Cladonia peziziformis*	45,312	0	0	0	0	1	0	0	0	0	0	0	0	0	0	0	2	15	3
*Cladonia rangiferina*	59,116	4	3	0	0	2	0	0	0	3	0	0	0	0	1	0	1	15	11
*Cladonia robbinsii*	50,467	1	2	0	0	0	0	0	0	1	0	0	0	0	1	0	1	15	4
*Cladonia stipitata*	60,062	5	4	0	0	2	0	0	0	1	0	0	0	0	0	0	1	15	9
*Cladonia subtenuis*	59,878	3	3	0	0	2	0	0	0	2	0	0	0	0	1	0	2	15	10
*Cladonia uncialis*	66,118	5	4	0	0	2	0	0	0	1	0	0	0	0	2	0	1	15	11
*Coccocarpia palmicola*	73,992	8	7	0	0	3	0	0	0	1	1	0	0	0	4	0	0	15	17
*Gomphillus americanus*	28,370	2	1	0	0	0	0	0	X	0	0	0	0	0	0	0	0	14	2
*Graphis lineola*	24,945	0	0	0	0	0	0	0	0	0	0	0	0	0	0	0	0	15	0
*Heterodermia albicans*	78,599	4	3	0	0	0	0	0	X	1	0	0	0	0	1	0	0	14	6
*Heterodermia appalachensis*	71,468	5	4	0	0	2	0	0	X	1	0	0	0	0	1	0	0	14	9
*Heterodermia casarettiana*	84,088	4	5	0	0	2	0	0	X	0	0	0	0	0	1	0	0	14	7
*Heterodermia echinata*	84,401	4	6	1	0	3	0	0	X	1	0	0	0	0	1	0	0	14	10
*Heterodermia speciosa1*	90,247	6	5	0	0	1	0	0	X	0	0	1	0	0	3	0	0	14	11
*Heterodermia speciosa2*	75,318	6	5	0	0	1	0	0	X	1	0	0	0	0	0	0	0	14	8
*Heterodermia squamulosa*	79,783	3	3	0	0	0	0	0	X	1	0	0	0	0	1	0	0	14	5
*Hypogymnia vittata*	38,888	0	0	0	0	0	0	0	X	1	0	0	0	0	0	0	1	14	2
*Icmadophila ericetorum*	43,773	0	0	0	0	1	0	0	0	1	0	0	0	0	1	0	0	15	3
*Imshaugia aleurites*	32,029	0	0	0	0	0	0	0	X	0	0	1	0	0	0	0	0	14	1
*Lecanora cinereofusca*	32,357	0	0	0	0	0	0	0	X	0	0	0	0	0	0	0	1	14	1
*Lecanora saxigena*	56,579	7	5	1	0	1	0	0	X	2	0	0	0	0	1	0	1	14	13
*Lepraria oxybapha*	40,221	0	0	0	0	1	0	0	0	0	0	0	0	0	2	0	1	15	4
*Leptogium hirsutum*	120,920	3	2	0	0	1	0	0	0	1	0	0	0	0	4	0	1	15	10
*Menegazzia subsimilis*	89,994	5	5	1	1	2	0	0	X	1	0	0	0	0	2	0	0	14	12
*Opegrapha vulgata*	38,937	1	0	0	0	0	0	0	0	0	0	0	1	0	0	0	1	15	3
*Parmotrema austrosinense*	92,711	6	4	0	1	0	0	0	X	2	0	0	1	0	3	0	0	14	13
*Parmotrema cetratum*	95,763	7	8	0	1	0	0	0	X	2	0	0	0	0	1	0	0	14	11
*Parmotrema crinitum*	86,310	5	4	0	0	2	0	0	X	2	0	0	1	0	2	0	1	14	13
*Parmotrema diffractaicum*	89,358	5	6	0	1	1	0	0	X	2	0	0	0	0	1	0	0	14	10
*Parmotrema internexum*	95,771	6	6	0	1	3	0	0	X	1	0	0	1	0	3	0	2	14	17
*Parmotrema margaritatum*	101,180	2	1	0	1	1	0	0	X	2	0	0	1	0	1	0	1	14	9
*Parmotrema mellissi*	97,183	6	5	0	0	2	0	0	X	2	0	0	1	0	3	0	1	14	15
*Parmotrema neotropicum*	124,067	6	6	0	2	2	0	0	X	7	1	0	1	0	3	1	0	14	23
*Parmotrema stuppeum*	108,024	5	6	0	1	2	0	0	X	3	0	0	0	0	2	0	0	14	13
*Parmotrema ultralucens*	79,456	1	1	0	1	1	0	0	X	2	0	0	1	0	2	0	0	14	8
*Pertusaria obruta*	86,976	6	0	0	0	3	0	0	0	1	0	0	0	0	2	0	0	15	13
*Pertusaria ostiolata*	62,290	1	2	0	0	3	0	0	0	0	0	0	0	0	1	0	0	15	5
*Pertusaria plittiana*	93,709	4	4	1	0	2	0	0	0	1	0	0	0	0	3	0	0	15	11
*Pertusaria propinqua*	79,765	5	3	1	0	2	0	0	0	1	0	0	0	0	2	0	0	15	11
*Phlyctis boliviensis*	25,319	0	0	0	0	0	0	0	0	0	0	0	0	0	0	0	0	15	0
*Phyllopsora corallina*	39,591	2	3	0	0	0	0	0	0	0	0	0	0	0	0	0	1	15	3
*Usnea ceratina*	65,539	8	0	0	0	3	1	0	X	2	0	0	0	0	2	0	1	14	17
*Usnea cornuta*	68,791	8	0	1	1	2	1	0	X	1	0	0	0	0	2	0	1	14	17
*Usnea halei*	82,851	7	3	1	1	5	1	0	X	2	0	0	0	0	1	0	0	14	18
*Usnea mutabilis*	61,314	2	1	2	1	3	1	0	X	1	0	0	0	0	2	0	1	14	13
*Usnea pensylvanica*	80,874	9	1	1	1	2	1	0	X	2	0	0	1	0	3	0	1	14	21
*Usnea subfusca*	52,486	4	1	1	1	2	0	0	X	1	0	0	0	0	1	0	0	14	10
*Usnea subgracilis*	95,033	7	0	2	1	3	0	0	X	1	0	0	0	0	3	0	0	14	17
*Usnea subscabrosa*	78,464	8	1	2	1	4	1	0	X	2	0	0	0	0	2	0	1	14	21

On average, the *cox1*gene contained the greatest number of introns within each genome (Table 1). As was the case overall, the number of introns (*R* ^2^ = 0.38, *p* = 0.004) and length of introns (*R* ^2^ = 0.53, *p* < 0.00001) within this gene were strongly correlated with genome size. However, after correcting for phylogenetic relatedness, the number of introns within the *cox1*gene was not significantly correlated with genome size (*p* = 0.69), suggesting phylogenetic signal in the number of *cox1*introns that was further evidenced by Blomberg's K (*p* = 0.00003) and Pagel's lambda (*p* = 0.014) values. The coding DNA sequence of the *cox1*gene was consistent in size across all the species examined and was not significantly correlated to overall genome size (Table 1; *R* ^2^ = 0.02, *p* = 0.885).

### 3.2 Synteny

The order of gene features was not consistent across all 58 genomes, suggesting some degree of gene‐block inversions and translocations. We examined six sets of congeners (i.e., members of a genus) and found conservation of gene order varied considerably even within genera. At one extreme, gene order was conserved for (a) all eight species of *Usnea*, (b) all but one of the 11 species of *Cladonia*, and (c) all but one of the seven species of *Heterodermia* (Supporting Information Figure S1). The exception in *Cladonia* was *C. uncialis*, which had an inversion of the block of genes containing “*nad6‐cox3‐mtLSU‐nad2‐nad3*.” The exception in *Heterodermia*was *H. echinata*, which featured a translocated *nad3*. In contrast, the two *Lecanora*species examined, which are closely related sister taxa (Lendemer & Harris, 2014), were markedly variable in both genome size and feature order (Supporting Information Figure S1; *L. cinereofusca* was 32,357 bp in length and *L. saxigena*was 56,579 bp in length). The ten *Parmotrema*species examined were syntenic with the exception of their *nad1*and *atp6*genes (Supporting Information Figure S1). In addition, two species (*P. austrosinense* and *P. stuppeum*) each contained two copies of *atp6*, one truncated and one full length; furthermore, these were the only mitochondrial genomes in this sample set to contain any duplication within the core set of protein‐coding genes (see Mycobiont Genome Content; Supporting Information Figure S1).

### 3.3 Phylogenetic relationships

Our alignment of rDNA and introns totaled 16,352 bp in length, and analyses of these data recovered the same overall genus level relationships found in prior large‐scale phylogenetic studies of the Lecanoromycetes (Miadlikowska et al., 2014). Phylogenetic relationships were in general well‐supported (PP = 1.0); however, seven nodes were not strongly supported (i.e., PP < 0.95 Figure 1). Percent pairwise divergence is reported for all 58 species (Supporting Information Table S2).

### 3.4 Homing endonucleases

Substantial numbers of ORFs with homology to homing endonucleases were present in the mitochondrial genomes we examined. Specifically, we identified two types of HEGs: LAGLIDADG and GIY‐YIG. These HEG elements, either full length or degenerated, were especially abundant in the introns of the *cox1*gene. The number of HEGs (Table 1; *R* ^2^ = 0.25, *p* = 0.06) and summed length of ORFs containing homing endonucleases (*R* ^2^ = 0.30, *p* = 0.021) were marginally correlated with genome size, and both remained significant after phylogenetic correction with PGLS (*p* = 0.04 and *p* = 0.04 respectively). The HEGs were present as either freestanding within an intron (identified by having unique start and stop codons) or fused/within the same reading frame as the intron it parasitized (identified as sharing a start or stop codon; Table 2). Twenty‐one samples (36%) contained instances of more than one HEG present within the same intron (*Cladonia caroliniana, C. furcata, C. rangiferina, C. robbinsii, C. stipitata, C. uncialis, Heterodermia albicans, H. casarettiana, H. echinata, H. speciosa1, H. speciosa2, Parmotrema cetratum, P. crinitum, P. diffractaicum, P. internexum, P. neotropicum, P. stuppeum, Pertusaria ostiolata, P. plittiana, Phyllopsora corallina,*and *Usnea halei*).

**Table 2:** Number of homing endonucleases (types LAGLIDADG and GIY‐YIG) within the *cox1*gene, number of HEGs freestanding within an intron, and number of HEGs fused and sharing the same reading frame as the *cox1*gene

Species	Number of *cox1*retrotransposons	Freestanding within an intron	Reading frame fused with the intron
*Alectoria fallacina*	6	3	3
*Arthonia ruana*	4	1	3
*Arthonia susa*	0	0	0
*Bacidia sp*.	0	0	0
*Cladonia apodocarpa*	3	0	3
*Cladonia caroliniana*	3	1	2
*Cladonia furcata*	3	1	2
*Cladonia leporina*	3	0	3
*Cladonia petrophila*	4	1	3
*Cladonia peziziformis*	0	0	0
*Cladonia rangiferina*	3	1	2
*Cladonia robbinsii*	2	1	1
*Cladonia stipitata*	4	1	3
*Cladonia subtenuis*	3	0	0
*Cladonia uncialis*	4	2	2
*Coccocarpia palmicola*	7	3	4
*Gomphillus americanus*	1	0	1
*Graphis lineola*	0	0	0
*Heterodermia albicans*	3	3	0
*Heterodermia appalachensis*	4	1	3
*Heterodermia casarettiana*	5	1	4
*Heterodermia echinata*	6	3	3
*Heterodermia speciosa1*	5	3	2
*Heterodermia speciosa2*	5	3	2
*Heterodermia squamulosa*	3	0	3
*Hypogymnia vittata*	0	0	0
*Icmadophila ericetorum*	0	0	0
*Imshaugia aleurites*	0	0	0
*Lecanora cinereofusca*	0	0	0
*Lecanora saxigena*	5	0	5
*Lepraria oxybapha*	0	0	0
*Leptogium hirsutum*	2	1	1
*Menegazzia subsimilis*	5	1	4
*Opegrapha vulgata*	0	0	0
*Parmotrema austrosinense*	4	1	3
*Parmotrema cetratum*	8	3	5
*Parmotrema crinitum*	4	2	2
*Parmotrema diffractaicum*	6	2	4
*Parmotrema internexum*	6	3	3
*Parmotrema margaritatum*	1	0	1
*Parmotrema mellissi*	5	3	2
*Parmotrema neotropicum*	6	4	2
*Parmotrema stuppeum*	6	3	3
*Parmotrema ultralucens*	1	0	1
*Pertusaria obruta*	0	0	0
*Pertusaria ostiolata*	2	1	1
*Pertusaria plittiana*	4	3	1
*Pertusaria propinqua*	3	1	2
*Phlyctis boliviensis*	0	0	0
*Phyllopsora corallina*	3	2	1
*Usnea ceratina*	0	0	0
*Usnea cornuta*	0	0	0
*Usnea halei*	3	0	3
*Usnea mutabilis*	1	0	1
*Usnea pensylvanica*	1	0	1
*Usnea subfusca*	1	0	1
*Usnea subgracilis*	0	0	0
*Usnea subscabrosa*	1	0	1

### 3.5 Intron gain and loss

Intron gain and loss were examined genome‐wide as well as specifically within the *cox1*gene. Ancestral state reconstruction indicated that, genome‐wide, the ancestral mitochondria of the species examined contained five to ten introns, with both subsequent gains and losses across the sample set. In the *cox1*gene, there were also an intermediate number of introns (3–5) that later underwent genus‐ and species‐specific gains and losses. Species of *Heterodermia, Parmotrema,*and *Usnea*showed overall trends toward intron gain (Table 1), with species of *Usnea*representing the most extreme case. However, based on the current sampling, we recovered species‐specific intron loss in each genus examined, with some species experiencing complete loss of introns within the *cox1*gene (*Arthonia ruana, Cladonia peziziformis, Graphis lineola*,* Hypogymnia vittata, Icmadophila ericetorum, Imshaugia aleurites,* *Lecanora cinereofusca, Lepraria oxybapha*, and *Phlyctis boliviensis*; Table 1 and Supporting Information Figure S2) as well as two species experiencing complete genome‐wide intron loss (*Graphis lineola*and *Phlyctis boliviensis*).

### 3.6 Transmission of intron sequences

Group I and group II introns can be transmitted both vertically and horizontally (Belfort & Bonocora, 2014; Cho, Qiu, Kuhlman, & Palmer, 1998; Goddard & Burt, 1999). Using ancestral state reconstructions, we inferred that the intron sequences which were represented more than once in the data set are vertically transmitted (Figure 4). However, for the unique introns in some species (introns colored black; Figure 1), we wished to determine where they had originated from (i.e., the nuclear mycobiont genome or the photobiont mitochondrial genome). To explore this further, we searched each of the 58 species meta‐assemblies for sequences with high similarity (>80%) to the introns extracted from the mycobiont mitochondrial genomes. We observed that the best hits were to contigs that had low sequence coverage (1–3×, which was the average coverage of the contigs associated with the mycobiont nuclear contigs in the assembly) and had sequence matches to fungal/lichen species in NCBI's nonredundant database (>80% identity and >60% coverage). This suggests that in these cases, the nuclear genome of the mycobiont may be acting as a potential reservoir from which mitochondrial introns can arise. The intron sequences were distributed in 19 clusters of two or more introns (Figure 3). Clusters 1, 3, 5–9, 11, and 13 were present in two or more genera suggesting a relatively early origin among sampled taxa in our tree, while clusters 2, 4, 10, 12, and 14–19 were present only within a single genus, suggesting more recent gains (Figure 4). Introns were more similar within a given genus (always >90% similarity) than between genera (>80% similarity, sometimes >90%), again suggesting ancestral gains and losses followed by subsequent mutations within a genus (Figure 2). Species of *Usnea* contained the highest number of introns that contained high (>80%) sequence similarity (*n* = 10; Figure 1) and accounted for four of the 19 clusters (Figure 3). While *Parmotrema* contained a large number of introns that were similar between species (*n* = 8), it also contained six unique introns found in only a subset of species and these were relatively derived within the genus (Figure 1).

### 3.7 Intron correlation to categorical data

Genome‐wide intron number was significantly and positively correlated with lichens that were cyanobacterial (*p*‐value = 0.00616), were macrolichens (*p*‐value = 0.0000873), and reproduce asexually (*p*‐value = 0.0306). Additionally, the number of introns present in *cox1*was significantly correlated with the macrolichen growth form (*p*‐value = 0.00034).

### 3.8 Divergence among *cox1*introns in *Usnea*

The *cox1*introns among *Usnea* were highly divergent in comparison with species in the other five genera for which multiple species were sampled. Species of *Usnea* also had on average the highest number of introns within the *cox1*gene, and these introns were generally short in length in comparison with other genera (Supporting Information Figure S2a). In addition, species of *Usnea*had the fewest number of parasitic homing endonucleases (Table 2).

## 4 DISCUSSION

In this study, we documented differences in the number and variability of introns within 21 genera of lichens (six of which we sampled more than one representative species) that are on par with the total variation present among major subdomains of the tree of life, such as metazoa, fungi, and plants. Previous research has demonstrated that intron number is variable between different species of nonlichenized Ascomycete fungi (e.g., *S. cerevisiae*is relatively intron‐poor in comparison with *Aspergillus nidulans;*Paquin et al., 1997; Nielsen et al., 2004) and can drive major differences in genome size in these organisms (Sandor, Zhang, & Xu, 2018). Our study recapitulates in lichenized fungi the pattern of dynamic intron gains and losses, even between sister species, and differences in genome size observed in other nonlichenized fungi (Figure 1) as well as comparing mitochondrial intron number and location among groups of closely related lichenized species. In these lichenized fungi, we recovered evidence for both genome size proliferation via intron gain and streamlining via loss of mitochondrial introns over a short evolutionary timescale. The striking examples in our dataset include sister species within a genus that in some cases differed by fivefold in intron number.

Across the mitochondrial genes present in lichen mycobionts, we found evidence for HEG element parasitism in 11 genes. Among these, *cox1*was by far the most heavily parasitized by LAGLIDADG and GIY‐YIG homing endonucleases, with 49 of the 58 species (~85%) containing at least one intron. Twenty‐one species contained two or more HEG elements in a single intron. This nested HEG arrangement has the potential to drive alternative splicing (Guha et al., 2017), which in some lineages may foster gene regulatory divergence under variable environmental conditions, as has been demonstrated in diatoms (Rastogi et al., 2018).

Ancestral state reconstruction revealed that *cox1*has undergone both intron gains and losses, the latter of which appear to be a derived feature, unique to multiple individual species in our dataset. The nine species for which no introns within *cox1*were detected (*Arthonia ruana, Cladonia peziziformis, Graphis lineola*,* Hypogymnia vittata, Icmadophila ericetorum, Imshaugia aleurites,* *Lecanora cinereofusca, Lepraria oxybapha*, and *Phlyctis boliviensis*) are characterized by substantial reductions in overall genome size and/or low overall numbers of introns across all mitochondrial genes (Table 1) and differ strikingly in these characteristics even compared to close congeners. These instances mark losses rather than gains and can be taken as evidence of parallel evolution across multiple, distantly related lichens. This evidence for parallel streamlining of mitochondrial genomes via loss of parasitic introns and HEG elements in these symbiotic organisms has been similarly documented at the level of coding genes (Pogoda et al., 2018). Curiously, the fact that these derived features were recovered only toward the tips of phylogenetic branches and never observed deeper in our phylogenetic tree suggests that complete intron loss is not evolutionarily stable in lichenized fungi.

The data presented here thus extend some evidence of genome streamlining in symbiomes (sensu Tripp et al., 2017) from protein‐coding genes to repetitive, noncoding elements (Andersson & Andersson, 1999; Hansen & Moran, 2014; Moran & Bennett, 2014; Nikoh et al., 2014; Pogoda et al., 2018), suggesting action of parallel selection throughout coding and noncoding portions of the mitochondrial genome. However, genome reduction has been accompanied by gains in genome size in several lineages (*Heterodermia, Parmotrema,*and *Usnea*), and reductions are neither ubiquitous nor the only mode of evolution across symbiotic lichenized fungi. This is similar to other fungal species (Paquin et al., 1997; Santamaria et al., 2009) and suggests that the lichen mycobiont mitochondrial genome is not stably undergoing genome streamlining via loss of intronic sequences.

Notably, some traits and lifestyle attributes of lichens sampled in this study correlate with intron number. Separately, macrolichens, lichens that have cyanobacterial photobionts, and/or lichens that reproduce asexually have significantly more introns than other species (see Intron Correlation to Categorical Data & Supporting Information Table S1). Macrolichen morphology is strongly correlated with asexuality (Tripp & Lendemer, ms in prep.). In asexually reproducing lichens, selection should be less effective at removing mildly deleterious mutations owing to processes such as Muller's ratchet (Haigh, 1978). Introns and other retrotransposable elements are expected to be slightly harmful, on average, due to the replication costs of their DNA and encoded RNA and proteins, and because by virtue of frequent replication and transposition throughout the genome, they have the capability of introducing harmful mutations within the host genome upon insertion (Cambareri et al., 1996; Nagy & Chandler, 2004). If the nuclear genome is indeed acting as a reservoir for these introns, asexual lichens will have a larger nuclear intron reservoir, due to the lack of recombination, than sexual lichens explaining why on average asexual lichens have more mitochondrial introns. Species that reproduce largely asexually also may have shorter overall generations times (Charlesworth & Charlesworth, 1997) and thus have more opportunities for selfish, parasitic elements such as introns to proliferate throughout their genomes. However, other studies have found that uniparental mitochondrial inheritance and the spread of HEGs may be influenced by certain mating type loci (Yan et al., 2018), thus suggesting that there are possible underlying genetic mechanisms that influence the spread of HEGs within the mitochondrial genome. Future work examining the presence/absence of HEGs, their spread throughout the genome, and the associated lichen mating types will help to further elucidate the underlying drivers of differences in intron number.

While introns do add noncoding length to genes, thus incurring costs during cell division and transcription, they offer the potential benefit of alternative splicing, contributing valuable flexibility in gene expression and regulation (Dibb, 1993; Jo & Choi, 2015; Lynch et al., 2006; Smith et al., 2018). Alternative splicing may in fact confer greater genetic flexibility to the mitochondrial genomes of plants and fungi compared to those of the relatively intron‐poor bilateral animals (Dibb, 1993; Jo & Choi, 2015; Kazan, 2003; Keren, Lev‐Maor, & Ast, 2010; Lynch et al., 2006). Future research exploring the transcription of mitochondrial genes in lichenized fungi may determine whether alternative splicing is occurring or whether the introns simply propagate because of faster generation times and/or reduced ability to eliminate these elements from genomes.

Our study recapitulates many of the patterns observed in nonlichenized fungi. We see relatively stable gene content with the notable exception of loss of mitochondrial *atp9*in some genera of lichenized fungi (Arthoniales; Bailey et al., ms in prep., Lecanorales, Ostropales and Teloschistales; Pogoda et al., 2018) and duplication of *atp6*in two species of *Parmotrema*(*P. austrosinense* and *P. stuppeum*). Nonlichenized fungi also maintain relatively stable gene content, for example, sometimes losing *nad1*(Sandor et al., 2018). Gene synteny is both maintained in some species and highly variable between others in both lichenized and nonlichenized fungi (Pogoda et al., 2018; Sandor et al., 2018). Additionally, genome size in both can vary widely, even between sister species, and is driven by often major differences in intron number, variable lengths of intergenic regions, and differences in the presence/absence of homing endonucleases (Pogoda et al., 2018). Our study adds to the growing literature on fungal mitochondria and demonstrates that lichenized fungi have many of the same polymorphisms of nonlichenized fungi.

### 4.1 A unique case of divergence within *Usnea*

*Usnea* (Old Man's Beard) is a morphologically distinctive and species‐rich lineage represented on every continent (Crespo et al., 2007). Speciation rates within *Usnea* have been estimated to be two to three times higher than rates in other members of Parmeliaceae (Kraichak et al., 2015). In this study, we found that species of *Usnea* harbored more variable intron sequences (i.e., sequence dissimilarity) compared to any other sampled genus (Figures 1 and 3). These data suggest a potential link between speciation rate and rate of intron evolution, potentially as a function of faster rates of mutation and/or faster generation times within *Usnea*.

Of further interest is our documentation that species of *Usnea* contained the fewest homing endonucleases parasitizing *cox1* introns despite containing a higher average number of shorter length introns (average summed intron sequence for *Usnea* = 4,200 bp, *Parmotrema* = 9,300 bp, *Heterodermia* = 7,700 bp) compared to any other genus in this study. The leading hypothesis to explain mechanisms of intron loss involves reverse transcription in which mRNAs are intermediately converted into cDNAs and the cDNAs, lacking some or all of the intronic sequences, participate in recombination to produce a gene sequence without introns (Roy & Gilbert, 2006; Zhang, Yang, & Niu, 2010). This process requires reverse transcription machinery such as reverse transcriptase, maturase, and homing endonucleases to be present (Roy & Gilbert, 2006; Zhang et al., 2010). Reconstruction of ancestral intron states in this study suggests that species of *Usnea* are marked by relatively recent gains of short intron sequences that have undergone species‐level losses. We suggest that these mitochondrial genomes have yet to be highly parasitized by HEG elements via vertical transmission and therefore lack some of the required reverse transcription machinery to excise introns, as other genera have likely acquired.

## 5 CONCLUSION

In this study, we explored both the genome‐wide intron landscape and dynamic evolution within *cox1*among numerous lichenized fungal mitochondrial genomes, demonstrating a high degree of parasitism of introns. These intronic elements are shared among varying levels of phylogenetic diversity: Some reflect sharing among different orders or classes separated by ~418 Ma years of evolution (e.g., *Cladonia*and *Arthonia;*Prieto & Wedin, 2013; Kumar, Stecher, Suleski, & Hedges, 2017; Cluster 3 in Figure 3), whereas others reflect sharing between only sister species. Our data show that intron gains and losses have occurred multiple times across the evolutionary history of the Lecanoromycetes, with substantial variability across the species examined.

Our data yielded evidence for nine instances of complete loss of introns within *cox1*and most other genes as well as two instances of complete, genome‐wide intron loss. This suggests that some (but not all) lichen mitochondrial genomes may be undergoing selection for genome streamlining via loss of repetitive, parasitic DNA elements, in a parallel manner to genome streamlining previously documented in coding regions of lichen mycobiont mitochondria. Our results suggest that asexual lichens accumulate introns faster than sexually reproducing taxa, and this may be due to shorter generation times and the effect of Muller's ratchet causing accumulation of mildly deleterious mutations.

Lichenized fungal mitochondria offer an important and unique system in which to study the evolution of these organelles in the context of an obligate symbiotic relationship, and our results highlight dynamism in intron gains and losses in these iconic and important symbiomes. Indeed, the amount of variability observed in lichens mirrors the differences otherwise documented between different subdomains (i.e., metazoans, plants, and fungi) across the tree of life. Continued exploration of a broader suite of lichen species may reveal further novel patterns as well as shed further light on those documented here. Additionally, exploring the lichen transcriptomes has the potential to illuminate the occurrence of alternative splicing and the impact it may have on lichen evolution.

## CONFLICT OF INTEREST

The authors have no competing/conflicts of interest to disclose.

## AUTHOR CONTRIBUTION

Cloe Pogoda conducted research, analyzed data, interpreted the data, and wrote the paper. Kyle Keepers conducted research and edited the paper. Dustin Bailey conducted research. Arif Nadiadi conducted research. James Lendemer collected samples, interpreted the data, and edited the paper. Erin Tripp collected samples, analyzed data, interpreted the data, and wrote the paper. Nolan Kane interpreted the data and edited the paper.

## Data Availability

DNA sequences: Genbank accessions MG711470, MH308713, MH015348, MH359412, MG958507, MG708277, MG711314, MG725377, MG941021, MG686615, KY460674, MG725618, MG851822, MG949117, KY352404, NC_034332, NC_034790, KY315996, MG733978, MG720574, MH359411, MG773606, KY328643, MG711806, MG964001, KY362374, KY124637, KY352227, MH359410, MH359409, KY348846, NC_034928, KY352491, KY315997, MG865664, MG799541, MG678039, MG642023, MG725341, MH243019, MG233922, MG754912, KY362439, MG807882, MG686614, KY346830, MG720572, MH359408, KY305663, NC_034779, KX987159, KY100278, MG722979, MG920803, KY321923, MG720812, MG720066, and MG720452. Final rDNA alignment uploaded to Zenodo #1420516. Sampling locations and morphological data: NYBG Herbarium http://sweetgum.nybg.org/science/vh/.
