# Supplementary material for: Genome streamlining via complete loss of introns has occurred multiple times in lichenized fungal mitochondria
Source: Ecol Evol. 2019 Mar 21;9(7):4245–63. doi: 10.1002/ece3.5056 (PMC6467859; doi:10.1002/ece3.5056)
Supplement: Supplementary file 1 [file ECE3-9-4245-s001.docx]

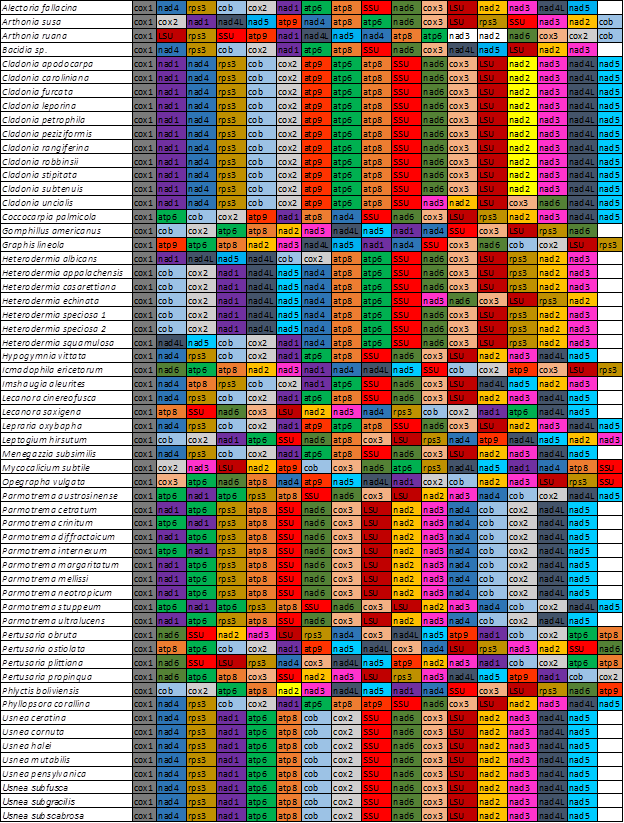


**Fig S1:** Gene order in mitochondrion of 58 lichenized fungal species. Species within a genus are grouped together. Each mitochondrion contained 14-15 genes with *atp9* absent in several genomes (i.e., *Alectoria fallacina*, *Bacidia* sp., *Gomphillus americanus*, *Heterodermia albicans, Heterodermia appalachensis, Heterodermia casarettiana, Heterodermia echinata, Heterodermia speciosa1, Heterodermia speciosa2, Heterodermia squamulose, Imshaugia aleurites, Lecanora cinereofusca, Lecanora saxigena, Menegazzia subsimilis, Parmotrema austrosinense, Parmotrema cetratum, Parmotrema crinitum, Parmotrema diffractaicum, Parmotrema internexum, Parmotrema margaritatum, Parmotrema mellissii, Parmotrema neotropicum, Parmotrema stuppeum , Parmotrema ultralucens, Usnea ceratina, Usnea cornuta, Usnea halei, Usnea mutabilis, Usnea pensylvanica, Usnea subfusca, Usnea subgracilis* and *Usnea subscabrosa*). Two species of *Parmotrema* (*P. austrosinense* and *P. stuppeum*) contained two copies of the *atp6* gene, one full length and presumably functional and the truncated and presumably non-functional. In twenty taxa (*Arthonia ruana, Arthonia susa, Coccocarpia palmicola, Gomphillus americanus, Graphis lineola, Heterodermia albicans, Heterodermia appalachensis, Heterodermia casarettiana, Heterodermia echinata, Heterodermia speciosa1, Heterodermia speciosa2, Heterodermia squamulose, Icmadophila ericetorum Leptogium hirsutum, Opegrapha vulgate, Pertusaria obruta, Pertusaria ostiolata, Pertusaria plittiana, Pertusaria propinqua* and *Phlyctis boliviensis*), *rps3* is encoded within an intron of the *mtLSU* gene, despite appearing in tandem in the figure.


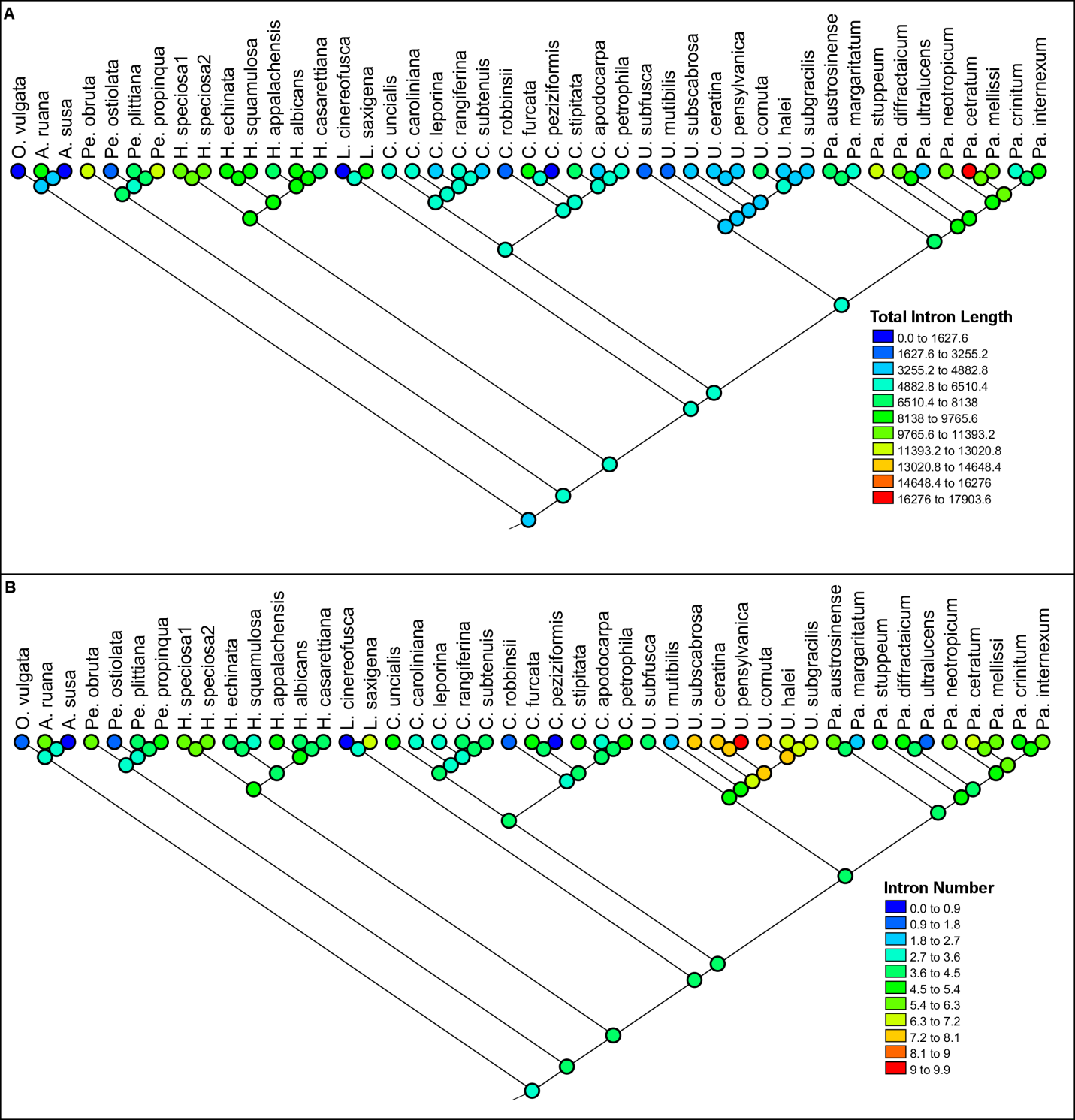


**Fig S2:** Ancestral state reconstruction for (A) total intron length of introns and (B) intron number in *cox1*. Pie colors at nodes reflect approximate intron content in ancestral lineages.


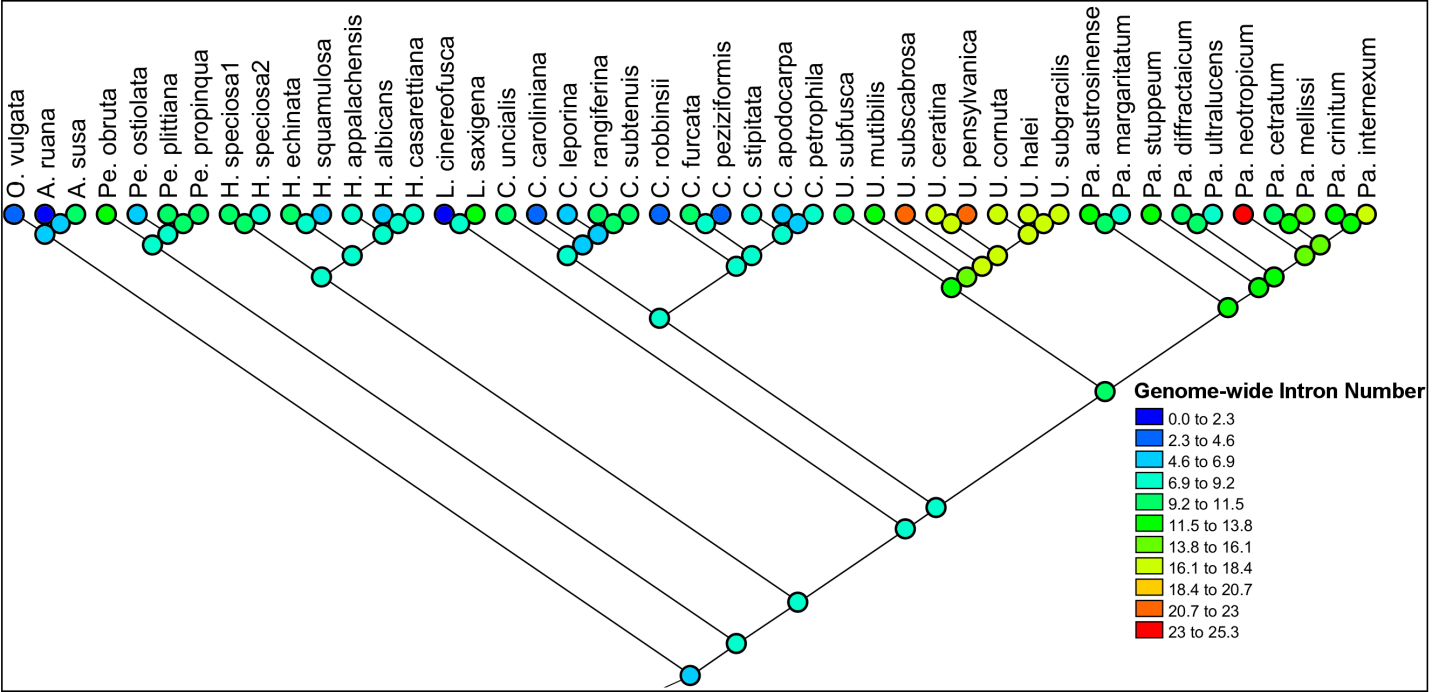


**Fig S3:** Ancestral state reconstruction for genome-wide intron number. Vertices are colored to indicate the approximate length/number of introns present in each species.
