# Supplementary material for: Genome streamlining via complete loss of introns has occurred multiple times in lichenized fungal mitochondria
Source: Ecol Evol. 2019 Mar 21;9(7):4245–63. doi: 10.1002/ece3.5056 (PMC6467859; doi:10.1002/ece3.5056)
Supplement: Supplementary file 2 [file ECE3-9-4245-s002.docx]

**Table S1:** Species name, collector and specimen voucher number, photobiont used, growth form and reproduction mode.

| **Species** | **Collector** | **Specimen Voucher #** | **Photobiont** | **Form** | **Reproduction Mode** | **GenBank Accession Number** |
| --- | --- | --- | --- | --- | --- | --- |
| Alectoria fallacina | Lendemer | 46255 | Green coccoid | Macrolichen | Asexual Lichenized | MG711470 |
| Arthonia ruana | Lendemer | 46304 | Green trentepohlia | Microlichen | Sexual | MH308713 |
| Arthonia susa | Lendemer | 45912 | Green trentepohlia | Microlichen | Sexual | MH015348 |
| Bacidia sp. | Lendemer | 48883 | Green trentepohlia | Microlichen | Asexual Lichenized | MH359412 |
| Cladonia apodocarpa | Lendemer | 48789 | Green coccoid | Macrolichen | Sexual | MG958507 |
| Cladonia caroliniana | Lendemer | 49894 | Green coccoid | Macrolichen | Sexual | MG708277 |
| Cladonia furcata | Lendemer | 49961 | Green coccoid | Macrolichen | Sexual | MG711314 |
| Cladonia leporina | Tripp | 6543 | Green coccoid | Macrolichen | Sexual | MG725377 |
| Cladonia petrophila | Lendemer | 55756 | Green coccoid | Macrolichen | Asexual Lichenized | MG941021 |
| Cladonia peziziformis | Lendemer | 45952 | Green coccoid | Macrolichen | Sexual | MG686615 |
| Cladonia rangiferina | Lendemer | 46392 | Green coccoid | Macrolichen | Sexual | KY460674 |
| Cladonia robbinsii | Lendemer | 49897 | Green coccoid | Macrolichen | Asexual Lichenized | MG725618 |
| Cladonia stipitata | Lendemer | 55210 | Green coccoid | Macrolichen | Sexual | MG851822 |
| Cladonia subtenuis | Lendemer | 49895 | Green coccoid | Macrolichen | Sexual | MG949117 |
| Cladonia uncialis | Lendemer | 46391 | Green coccoid | Macrolichen | Sexual | KY352404 |
| Coccocarpia palmicola | Lendemer | 45953 | Cyanobacterium | Macrolichen | Asexual Lichenized | NC 034332 |
| Gomphillus americanus | Lendemer | 45954 | Green trentepohlia | Microlichen | Sexual | NC 034790 |
| Graphis lineola | Lendemer | 46096 | Green trentepohlia | Microlichen | Sexual | KY315996 |
| Heterodermia albicans | Lendemer | 49538 | Green coccoid | Macrolichen | Asexual Lichenized | MG733978 |
| Heterodermia appalachensis | Lendemer | 46928 | Green coccoid | Macrolichen | Asexual Lichenized | MG720574 |
| Heterodermia casarettiana | Lendemer | 46038 | Green coccoid | Macrolichen | Asexual Lichenized | MH359411 |
| Heterodermia echinata | Tripp | 6430 | Green coccoid | Macrolichen | Sexual | MG773606 |
| Heterodermia speciosa1 | Lendemer | 46925 | Green coccoid | Macrolichen | Asexual Lichenized | KY328643 |
| Heterodermia speciosa2 | Lendemer | 49524 | Green coccoid | Macrolichen | Asexual Lichenized | MG711806 |
| Heterodermia squamulosa | Tripp | 5516 | Green coccoid | Macrolichen | Asexual Lichenized | MG964001 |
| Hypogymnia vittata | Lendemer | 46170 | Green coccoid | Macrolichen | Asexual Lichenized | KY362374 |
| Icmadophila ericetorum | Lendemer | 46191 | Green coccoid | Microlichen | Sexual | KY124637 |
| Imshaugia aleurites | Lendemer | 46246 | Green coccoid | Macrolichen | Asexual Lichenized | KY352227 |
| Lecanora cinereofusca | Lendemer | 45937 | Green coccoid | Microlichen | Sexual | MH359410 |
| Lecanora saxigena | Lendemer | 49057 | Green coccoid | Microlichen | Sexual | MH359409 |
| Lepraria oxybapha | Lendemer | 46299 | Green coccoid | Microlichen | Asexual Lichenized | KY348846 |
| Leptogium hirsutum | Lendemer | 46937 | Cyanobacterium | Macrolichen | Asexual Lichenized | NC 034928 |
| Menegazzia subsimilis | Lendemer | 46149 | Green coccoid | Macrolichen | Asexual Lichenized | KY352491 |
| Opegrapha vulgata | Lendemer | 45910 | Green trentepohlia | Microlichen | Sexual | KY315997 |
| Parmotrema austrosinense | Lendemer | 49663 | Green coccoid | Macrolichen | Asexual Lichenized | MG865664 |
| Parmotrema cetratum | Lendemer | 46914 | Green coccoid | Macrolichen | Sexual | MG799541 |
| Parmotrema crinitum | Tripp | 5527 | Green coccoid | Macrolichen | Asexual Lichenized | MG678039 |
| Parmotrema diffractaicum | Lendemer | 45986 | Green coccoid | Macrolichen | Asexual Lichenized | MG642023 |
| Parmotrema internexum | Lendemer | 49587 | Green coccoid | Macrolichen | Asexual Lichenized | MG725341 |
| Parmotrema margaritatum | Lendemer | 44568 | Green coccoid | Macrolichen | Asexual Lichenized | MH243019 |
| Parmotrema mellissi | Tripp | 3909 | Green coccoid | Macrolichen | Asexual Lichenized | MG233922 |
| Parmotrema neotropicum | Lendemer | 44839 | Green coccoid | Macrolichen | Asexual Lichenized | MG754912 |
| Parmotrema stuppeum | Lendemer | 46478 | Green coccoid | Macrolichen | Asexual Lichenized | KY362439 |
| Parmotrema ultralucens | Tripp | 6176 | Green coccoid | Macrolichen | Asexual Lichenized | MG807882 |
| Pertusaria obruta | Lendemer | 49548 | Green coccoid | Microlichen | Sexual | MG686614 |
| Pertusaria ostiolata | Lendemer | 51850 | Green coccoid | Microlichen | Sexual | KY346830 |
| Pertusaria plittiana | Tripp | 5465 | Green coccoid | Microlichen | Sexual | MG720572 |
| Pertusaria propinqua | Lendemer | 48825 | Green coccoid | Microlichen | Sexual | MH359408 |
| Phlyctis boliviensis | Tripp | 6443 | Green coccoid | Microlichen | Asexual Lichenized | KY305663 |
| Phyllopsora corallina | Lendemer | 45963 | Green coccoid | Microlichen | Asexual Lichenized | NC 034779 |
| Usnea ceratina | Lendemer | 46119 | Green coccoid | Macrolichen | Asexual Lichenized | KX987159 |
| Usnea cornuta | Lendemer | 46118 | Green coccoid | Macrolichen | Asexual Lichenized | KY100278 |
| Usnea halei | Lendemer | 46374 | Green coccoid | Macrolichen | Asexual Lichenized | MG722979 |
| Usnea mutabilis | Lendemer | 49260 | Green coccoid | Macrolichen | Asexual Lichenized | MG920803 |
| Usnea pensylvanica | Lendemer | 46091 | Green coccoid | Macrolichen | Asexual Lichenized | KY321923 |
| Usnea subfusca | Lendemer | 46309 | Green coccoid | Macrolichen | Sexual | MG720812 |
| Usnea subgracilis | Lendemer | 48717 | Green coccoid | Macrolichen | Asexual Lichenized | MG720066 |
| Usnea subscabrosa | Lendemer | 50256 | Green coccoid | Macrolichen | Asexual Lichenized | MG720452 |

**Table S2:** Percent pairwise divergence is reported for all 58 species.

| Alectoria_fallacina |  |  |  |  |  |  |  |  |  |  |  |  |  |  |  |  |  |  |  |  |  |  |  |  |  |  |  |  |  |  |  |  |  |  |  |  |  |  |  |  |  |  |  |  |  |  |  |  |  |  |  |  |  |  |  |  |  |
| --- | --- | --- | --- | --- | --- | --- | --- | --- | --- | --- | --- | --- | --- | --- | --- | --- | --- | --- | --- | --- | --- | --- | --- | --- | --- | --- | --- | --- | --- | --- | --- | --- | --- | --- | --- | --- | --- | --- | --- | --- | --- | --- | --- | --- | --- | --- | --- | --- | --- | --- | --- | --- | --- | --- | --- | --- | --- |
| Arthonia_ruana | 0.10588 |  |  |  |  |  |  |  |  |  |  |  |  |  |  |  |  |  |  |  |  |  |  |  |  |  |  |  |  |  |  |  |  |  |  |  |  |  |  |  |  |  |  |  |  |  |  |  |  |  |  |  |  |  |  |  |  |
| Arthonia_susa | 0.12832 | 0.08006 |  |  |  |  |  |  |  |  |  |  |  |  |  |  |  |  |  |  |  |  |  |  |  |  |  |  |  |  |  |  |  |  |  |  |  |  |  |  |  |  |  |  |  |  |  |  |  |  |  |  |  |  |  |  |  |
| Bacidia_sp | 0.07081 | 0.11755 | 0.14606 |  |  |  |  |  |  |  |  |  |  |  |  |  |  |  |  |  |  |  |  |  |  |  |  |  |  |  |  |  |  |  |  |  |  |  |  |  |  |  |  |  |  |  |  |  |  |  |  |  |  |  |  |  |  |
| Cladonia_apodocarpa | 0.03074 | 0.10395 | 0.12584 | 0.06984 |  |  |  |  |  |  |  |  |  |  |  |  |  |  |  |  |  |  |  |  |  |  |  |  |  |  |  |  |  |  |  |  |  |  |  |  |  |  |  |  |  |  |  |  |  |  |  |  |  |  |  |  |  |
| Cladonia_caroliniana | 0.02928 | 0.10515 | 0.12629 | 0.06831 | 0.00666 |  |  |  |  |  |  |  |  |  |  |  |  |  |  |  |  |  |  |  |  |  |  |  |  |  |  |  |  |  |  |  |  |  |  |  |  |  |  |  |  |  |  |  |  |  |  |  |  |  |  |  |  |
| Cladonia_furcata | 0.03256 | 0.10779 | 0.12939 | 0.07185 | 0.00455 | 0.00984 |  |  |  |  |  |  |  |  |  |  |  |  |  |  |  |  |  |  |  |  |  |  |  |  |  |  |  |  |  |  |  |  |  |  |  |  |  |  |  |  |  |  |  |  |  |  |  |  |  |  |  |
| Cladonia_leporina | 0.03002 | 0.10405 | 0.12636 | 0.06832 | 0.00877 | 0.00666 | 0.01125 |  |  |  |  |  |  |  |  |  |  |  |  |  |  |  |  |  |  |  |  |  |  |  |  |  |  |  |  |  |  |  |  |  |  |  |  |  |  |  |  |  |  |  |  |  |  |  |  |  |  |
| Cladonia_petrophila | 0.03108 | 0.10476 | 0.12666 | 0.0698 | 0.0014 | 0.00665 | 0.00455 | 0.00876 |  |  |  |  |  |  |  |  |  |  |  |  |  |  |  |  |  |  |  |  |  |  |  |  |  |  |  |  |  |  |  |  |  |  |  |  |  |  |  |  |  |  |  |  |  |  |  |  |  |
| Cladonia_peziziformis | 0.03182 | 0.10773 | 0.12895 | 0.07297 | 0.00455 | 0.00913 | 0.00315 | 0.01125 | 0.00455 |  |  |  |  |  |  |  |  |  |  |  |  |  |  |  |  |  |  |  |  |  |  |  |  |  |  |  |  |  |  |  |  |  |  |  |  |  |  |  |  |  |  |  |  |  |  |  |  |
| Cladonia_rangiferina | 0.02929 | 0.10402 | 0.12555 | 0.06755 | 0.00701 | 0.0042 | 0.00913 | 0.00525 | 0.007 | 0.00877 |  |  |  |  |  |  |  |  |  |  |  |  |  |  |  |  |  |  |  |  |  |  |  |  |  |  |  |  |  |  |  |  |  |  |  |  |  |  |  |  |  |  |  |  |  |  |  |
| Cladonia_robbinsii | 0.03037 | 0.10481 | 0.12586 | 0.06946 | 0.0021 | 0.00666 | 0.00526 | 0.00947 | 0.0021 | 0.00525 | 0.0063 |  |  |  |  |  |  |  |  |  |  |  |  |  |  |  |  |  |  |  |  |  |  |  |  |  |  |  |  |  |  |  |  |  |  |  |  |  |  |  |  |  |  |  |  |  |  |
| Cladonia_stipitata | 0.0311 | 0.10482 | 0.12674 | 0.06868 | 0.0014 | 0.00666 | 0.0042 | 0.00877 | 0.0014 | 0.00455 | 0.00701 | 0.0021 |  |  |  |  |  |  |  |  |  |  |  |  |  |  |  |  |  |  |  |  |  |  |  |  |  |  |  |  |  |  |  |  |  |  |  |  |  |  |  |  |  |  |  |  |  |
| Cladonia_subtenuis | 0.02858 | 0.10446 | 0.1256 | 0.06682 | 0.00842 | 0.00525 | 0.01055 | 0.00631 | 0.00842 | 0.01019 | 0.00175 | 0.00772 | 0.00842 |  |  |  |  |  |  |  |  |  |  |  |  |  |  |  |  |  |  |  |  |  |  |  |  |  |  |  |  |  |  |  |  |  |  |  |  |  |  |  |  |  |  |  |  |
| Cladonia_uncialis | 0.03036 | 0.1044 | 0.1255 | 0.06637 | 0.00595 | 0.0056 | 0.00842 | 0.00701 | 0.00595 | 0.00842 | 0.00455 | 0.00595 | 0.00525 | 0.00596 |  |  |  |  |  |  |  |  |  |  |  |  |  |  |  |  |  |  |  |  |  |  |  |  |  |  |  |  |  |  |  |  |  |  |  |  |  |  |  |  |  |  |  |
| Coccocarpia_palmicola | 0.07861 | 0.10808 | 0.12136 | 0.10178 | 0.08473 | 0.0847 | 0.08673 | 0.08398 | 0.08468 | 0.0859 | 0.08359 | 0.08511 | 0.08512 | 0.08362 | 0.08276 |  |  |  |  |  |  |  |  |  |  |  |  |  |  |  |  |  |  |  |  |  |  |  |  |  |  |  |  |  |  |  |  |  |  |  |  |  |  |  |  |  |  |
| Gomphillus_americanus | 0.1669 | 0.21368 | 0.2386 | 0.17888 | 0.16274 | 0.16196 | 0.16484 | 0.16146 | 0.16276 | 0.1656 | 0.16117 | 0.16276 | 0.16279 | 0.16305 | 0.16329 | 0.20949 |  |  |  |  |  |  |  |  |  |  |  |  |  |  |  |  |  |  |  |  |  |  |  |  |  |  |  |  |  |  |  |  |  |  |  |  |  |  |  |  |  |
| Graphis_lineola | 0.13191 | 0.17055 | 0.20068 | 0.14495 | 0.13311 | 0.13142 | 0.1358 | 0.13488 | 0.13313 | 0.13573 | 0.13276 | 0.13401 | 0.13402 | 0.13281 | 0.13439 | 0.16827 | 0.17671 |  |  |  |  |  |  |  |  |  |  |  |  |  |  |  |  |  |  |  |  |  |  |  |  |  |  |  |  |  |  |  |  |  |  |  |  |  |  |  |  |
| Heterodermia_albicans | 0.06987 | 0.13549 | 0.15284 | 0.09261 | 0.06745 | 0.06705 | 0.07022 | 0.0682 | 0.06704 | 0.06786 | 0.06822 | 0.06782 | 0.06783 | 0.06786 | 0.06744 | 0.11159 | 0.18194 | 0.14121 |  |  |  |  |  |  |  |  |  |  |  |  |  |  |  |  |  |  |  |  |  |  |  |  |  |  |  |  |  |  |  |  |  |  |  |  |  |  |  |
| Heterodermia_appalachensis | 0.06397 | 0.12635 | 0.14572 | 0.08566 | 0.06158 | 0.06041 | 0.0639 | 0.06345 | 0.06192 | 0.06159 | 0.06272 | 0.06195 | 0.06196 | 0.06391 | 0.06195 | 0.10243 | 0.18119 | 0.14105 | 0.01984 |  |  |  |  |  |  |  |  |  |  |  |  |  |  |  |  |  |  |  |  |  |  |  |  |  |  |  |  |  |  |  |  |  |  |  |  |  |  |
| Heterodermia_casarettiana | 0.06471 | 0.12555 | 0.14441 | 0.0853 | 0.05963 | 0.06076 | 0.06119 | 0.06152 | 0.05997 | 0.05964 | 0.06079 | 0.06 | 0.06 | 0.06197 | 0.06002 | 0.10123 | 0.178 | 0.14064 | 0.01874 | 0.01019 |  |  |  |  |  |  |  |  |  |  |  |  |  |  |  |  |  |  |  |  |  |  |  |  |  |  |  |  |  |  |  |  |  |  |  |  |  |
| Heterodermia_echinata | 0.06362 | 0.12725 | 0.14666 | 0.08411 | 0.06196 | 0.06234 | 0.06427 | 0.06385 | 0.0623 | 0.06197 | 0.06312 | 0.06232 | 0.06233 | 0.06431 | 0.06235 | 0.10092 | 0.18052 | 0.1364 | 0.02127 | 0.01339 | 0.01054 |  |  |  |  |  |  |  |  |  |  |  |  |  |  |  |  |  |  |  |  |  |  |  |  |  |  |  |  |  |  |  |  |  |  |  |  |
| Heterodermia_speciosa1 | 0.06515 | 0.12696 | 0.14496 | 0.08653 | 0.06086 | 0.06271 | 0.06317 | 0.06422 | 0.0612 | 0.06084 | 0.06349 | 0.06045 | 0.06123 | 0.06468 | 0.06197 | 0.10171 | 0.18112 | 0.14103 | 0.0191 | 0.01303 | 0.00877 | 0.01303 |  |  |  |  |  |  |  |  |  |  |  |  |  |  |  |  |  |  |  |  |  |  |  |  |  |  |  |  |  |  |  |  |  |  |  |
| Heterodermia_speciosa2 | 0.06515 | 0.12696 | 0.14496 | 0.08653 | 0.06086 | 0.06271 | 0.06317 | 0.06422 | 0.0612 | 0.06084 | 0.06349 | 0.06045 | 0.06123 | 0.06468 | 0.06197 | 0.10171 | 0.18112 | 0.14103 | 0.0191 | 0.01303 | 0.00877 | 0.01303 | 0 |  |  |  |  |  |  |  |  |  |  |  |  |  |  |  |  |  |  |  |  |  |  |  |  |  |  |  |  |  |  |  |  |  |  |
| Heterodermia_squamulosa | 0.06245 | 0.12508 | 0.14314 | 0.08447 | 0.05775 | 0.05813 | 0.05931 | 0.05889 | 0.05809 | 0.05776 | 0.0589 | 0.05812 | 0.05812 | 0.05934 | 0.05888 | 0.10123 | 0.17999 | 0.14016 | 0.01804 | 0.01019 | 0.00771 | 0.00983 | 0.01019 | 0.01019 |  |  |  |  |  |  |  |  |  |  |  |  |  |  |  |  |  |  |  |  |  |  |  |  |  |  |  |  |  |  |  |  |  |
| Hypogymnia_vittata | 0.01374 | 0.10902 | 0.13065 | 0.06914 | 0.03033 | 0.03032 | 0.02998 | 0.03106 | 0.03067 | 0.02996 | 0.02997 | 0.02996 | 0.03069 | 0.02998 | 0.02888 | 0.08322 | 0.1689 | 0.13649 | 0.07025 | 0.06472 | 0.06353 | 0.06283 | 0.06399 | 0.06399 | 0.06203 |  |  |  |  |  |  |  |  |  |  |  |  |  |  |  |  |  |  |  |  |  |  |  |  |  |  |  |  |  |  |  |  |
| Icmadophila_ericetorum | 0.08328 | 0.13141 | 0.15489 | 0.10641 | 0.08309 | 0.08236 | 0.08715 | 0.08236 | 0.0831 | 0.08634 | 0.08163 | 0.08153 | 0.08392 | 0.08199 | 0.08352 | 0.11681 | 0.1729 | 0.14449 | 0.1003 | 0.09501 | 0.09377 | 0.0938 | 0.09587 | 0.09587 | 0.09533 | 0.08797 |  |  |  |  |  |  |  |  |  |  |  |  |  |  |  |  |  |  |  |  |  |  |  |  |  |  |  |  |  |  |  |
| Imshaugia_aleurites | 0.01338 | 0.10947 | 0.13201 | 0.06846 | 0.03036 | 0.02962 | 0.03217 | 0.03037 | 0.0307 | 0.03107 | 0.03 | 0.03071 | 0.02961 | 0.02927 | 0.02961 | 0.08284 | 0.16602 | 0.12979 | 0.0698 | 0.06311 | 0.06272 | 0.06239 | 0.06354 | 0.06354 | 0.06123 | 0.01515 | 0.08475 |  |  |  |  |  |  |  |  |  |  |  |  |  |  |  |  |  |  |  |  |  |  |  |  |  |  |  |  |  |  |
| Lecanora_cinereofusca | 0.04072 | 0.11199 | 0.13431 | 0.07323 | 0.03509 | 0.03287 | 0.03806 | 0.03581 | 0.03507 | 0.03804 | 0.03397 | 0.03545 | 0.03545 | 0.0351 | 0.03434 | 0.08597 | 0.17074 | 0.14064 | 0.06458 | 0.06108 | 0.06102 | 0.06218 | 0.06187 | 0.06187 | 0.0599 | 0.04067 | 0.08648 | 0.04326 |  |  |  |  |  |  |  |  |  |  |  |  |  |  |  |  |  |  |  |  |  |  |  |  |  |  |  |  |  |
| Lecanora_saxigena | 0.04267 | 0.11639 | 0.13753 | 0.08268 | 0.04186 | 0.0411 | 0.04484 | 0.0415 | 0.04184 | 0.04408 | 0.04076 | 0.04222 | 0.04223 | 0.04225 | 0.04111 | 0.08779 | 0.17467 | 0.14044 | 0.07818 | 0.07296 | 0.07446 | 0.07493 | 0.07573 | 0.07573 | 0.07256 | 0.04747 | 0.08846 | 0.04677 | 0.04409 |  |  |  |  |  |  |  |  |  |  |  |  |  |  |  |  |  |  |  |  |  |  |  |  |  |  |  |  |
| Lepraria_oxybapha | 0.03077 | 0.1033 | 0.12342 | 0.07256 | 0.02091 | 0.0216 | 0.0227 | 0.02268 | 0.02125 | 0.02125 | 0.0216 | 0.02198 | 0.02127 | 0.02197 | 0.02197 | 0.08167 | 0.16516 | 0.13582 | 0.0691 | 0.06356 | 0.0643 | 0.06437 | 0.06326 | 0.06326 | 0.06205 | 0.03253 | 0.08348 | 0.03365 | 0.03737 | 0.04154 |  |  |  |  |  |  |  |  |  |  |  |  |  |  |  |  |  |  |  |  |  |  |  |  |  |  |  |
| Leptogium_hirsutum | 0.10307 | 0.11984 | 0.1236 | 0.12445 | 0.10881 | 0.10832 | 0.11008 | 0.10675 | 0.10874 | 0.11045 | 0.10681 | 0.10918 | 0.10881 | 0.10551 | 0.10598 | 0.0873 | 0.22711 | 0.18697 | 0.12743 | 0.12008 | 0.12011 | 0.12177 | 0.12141 | 0.12141 | 0.11885 | 0.10635 | 0.13885 | 0.10792 | 0.1106 | 0.11065 | 0.10356 |  |  |  |  |  |  |  |  |  |  |  |  |  |  |  |  |  |  |  |  |  |  |  |  |  |  |
| Menegazzia_subsimilis | 0.02091 | 0.10629 | 0.13124 | 0.07289 | 0.03367 | 0.03439 | 0.03625 | 0.03404 | 0.03402 | 0.03551 | 0.03295 | 0.03331 | 0.03404 | 0.03369 | 0.0333 | 0.08139 | 0.17283 | 0.13327 | 0.07144 | 0.06478 | 0.06438 | 0.06446 | 0.06522 | 0.06522 | 0.06438 | 0.02055 | 0.08379 | 0.02055 | 0.04481 | 0.04647 | 0.03628 | 0.1093 |  |  |  |  |  |  |  |  |  |  |  |  |  |  |  |  |  |  |  |  |  |  |  |  |  |
| Opegrapha_vulgata | 0.09929 | 0.10278 | 0.12652 | 0.12533 | 0.09931 | 0.1005 | 0.1035 | 0.09886 | 0.09932 | 0.10303 | 0.09933 | 0.10015 | 0.10017 | 0.10022 | 0.09892 | 0.12615 | 0.20845 | 0.18608 | 0.12331 | 0.11683 | 0.11407 | 0.11729 | 0.11614 | 0.11614 | 0.11441 | 0.102 | 0.13335 | 0.10491 | 0.10683 | 0.11628 | 0.10522 | 0.12859 | 0.10254 |  |  |  |  |  |  |  |  |  |  |  |  |  |  |  |  |  |  |  |  |  |  |  |  |
| Parmotrema_austrosinense | 0.0148 | 0.10857 | 0.12937 | 0.06878 | 0.03218 | 0.03144 | 0.03438 | 0.03181 | 0.03252 | 0.03364 | 0.03218 | 0.03253 | 0.03254 | 0.03219 | 0.03253 | 0.0832 | 0.16203 | 0.13211 | 0.06791 | 0.06166 | 0.0609 | 0.06094 | 0.06209 | 0.06209 | 0.0594 | 0.01692 | 0.0832 | 0.01693 | 0.04365 | 0.04977 | 0.03366 | 0.10716 | 0.01802 | 0.09802 |  |  |  |  |  |  |  |  |  |  |  |  |  |  |  |  |  |  |  |  |  |  |  |
| Parmotrema_cetratum | 0.01197 | 0.10944 | 0.13109 | 0.06922 | 0.03111 | 0.03037 | 0.03256 | 0.03074 | 0.03145 | 0.03183 | 0.03039 | 0.03074 | 0.03147 | 0.0304 | 0.03146 | 0.08161 | 0.16384 | 0.13309 | 0.06796 | 0.06287 | 0.06286 | 0.06215 | 0.0633 | 0.0633 | 0.06061 | 0.01337 | 0.08326 | 0.01409 | 0.04181 | 0.04754 | 0.03257 | 0.10876 | 0.01731 | 0.1 | 0.00631 |  |  |  |  |  |  |  |  |  |  |  |  |  |  |  |  |  |  |  |  |  |  |
| Parmotrema_crinitum | 0.01338 | 0.11031 | 0.13199 | 0.06961 | 0.03183 | 0.0311 | 0.03293 | 0.03147 | 0.03218 | 0.03255 | 0.03111 | 0.03147 | 0.0322 | 0.03113 | 0.03219 | 0.08319 | 0.16429 | 0.13351 | 0.06722 | 0.06213 | 0.06212 | 0.06141 | 0.06256 | 0.06256 | 0.05986 | 0.01373 | 0.08446 | 0.01551 | 0.04181 | 0.04755 | 0.03221 | 0.1104 | 0.01803 | 0.10084 | 0.00772 | 0.0028 |  |  |  |  |  |  |  |  |  |  |  |  |  |  |  |  |  |  |  |  |  |
| Parmotrema_diffractaicum | 0.01266 | 0.10852 | 0.13136 | 0.06878 | 0.03036 | 0.02889 | 0.03181 | 0.03145 | 0.0307 | 0.03107 | 0.0289 | 0.02999 | 0.03072 | 0.02927 | 0.03071 | 0.08197 | 0.16245 | 0.12886 | 0.07063 | 0.0651 | 0.06508 | 0.06438 | 0.06478 | 0.06478 | 0.06283 | 0.01407 | 0.08279 | 0.01408 | 0.04215 | 0.04823 | 0.03218 | 0.10904 | 0.01873 | 0.10115 | 0.00771 | 0.00385 | 0.00595 |  |  |  |  |  |  |  |  |  |  |  |  |  |  |  |  |  |  |  |  |
| Parmotrema_internexum | 0.01696 | 0.11412 | 0.13464 | 0.07358 | 0.03626 | 0.0348 | 0.037 | 0.03517 | 0.03661 | 0.03699 | 0.03481 | 0.0359 | 0.03663 | 0.03519 | 0.03589 | 0.08609 | 0.16327 | 0.13337 | 0.06791 | 0.06248 | 0.06246 | 0.06176 | 0.0629 | 0.0629 | 0.06021 | 0.01767 | 0.08687 | 0.01765 | 0.04558 | 0.05099 | 0.03628 | 0.11217 | 0.02164 | 0.10458 | 0.01161 | 0.00561 | 0.00526 | 0.00878 |  |  |  |  |  |  |  |  |  |  |  |  |  |  |  |  |  |  |  |
| Parmotrema_margaritatum | 0.02633 | 0.11318 | 0.13495 | 0.07505 | 0.04325 | 0.04438 | 0.04588 | 0.04329 | 0.04474 | 0.04586 | 0.04367 | 0.04403 | 0.04476 | 0.04408 | 0.04475 | 0.09066 | 0.16951 | 0.13128 | 0.07492 | 0.06889 | 0.06812 | 0.06742 | 0.06857 | 0.06857 | 0.06586 | 0.02848 | 0.08913 | 0.02994 | 0.05381 | 0.06038 | 0.04552 | 0.11252 | 0.03033 | 0.10367 | 0.02055 | 0.01768 | 0.01911 | 0.02053 | 0.02344 |  |  |  |  |  |  |  |  |  |  |  |  |  |  |  |  |  |  |
| Parmotrema_mellissi | 0.01196 | 0.10929 | 0.1301 | 0.06913 | 0.03107 | 0.03034 | 0.03327 | 0.0307 | 0.03141 | 0.03253 | 0.03035 | 0.0307 | 0.03143 | 0.03036 | 0.03142 | 0.08162 | 0.16276 | 0.13129 | 0.06792 | 0.06204 | 0.06203 | 0.06132 | 0.06247 | 0.06247 | 0.05978 | 0.01407 | 0.08276 | 0.01479 | 0.04252 | 0.04862 | 0.03327 | 0.10948 | 0.0173 | 0.09952 | 0.0056 | 0.0028 | 0.0042 | 0.00419 | 0.00772 | 0.0191 |  |  |  |  |  |  |  |  |  |  |  |  |  |  |  |  |  |
| Parmotrema_neotropicum | 0.01232 | 0.10821 | 0.12935 | 0.06916 | 0.03146 | 0.03072 | 0.03292 | 0.03183 | 0.0318 | 0.03218 | 0.03146 | 0.03182 | 0.03182 | 0.03147 | 0.03181 | 0.08247 | 0.16513 | 0.13142 | 0.06989 | 0.06476 | 0.06437 | 0.06404 | 0.06519 | 0.06519 | 0.06249 | 0.01372 | 0.08323 | 0.01373 | 0.04182 | 0.0479 | 0.03292 | 0.10801 | 0.01983 | 0.10006 | 0.00842 | 0.0049 | 0.00701 | 0.00525 | 0.00985 | 0.02091 | 0.00595 |  |  |  |  |  |  |  |  |  |  |  |  |  |  |  |  |
| Parmotrema_stuppeum | 0.0109 | 0.10744 | 0.12944 | 0.06728 | 0.02928 | 0.02927 | 0.03074 | 0.02965 | 0.02963 | 0.03072 | 0.02856 | 0.02892 | 0.02964 | 0.02857 | 0.02964 | 0.08121 | 0.16422 | 0.13097 | 0.06837 | 0.06324 | 0.06247 | 0.06251 | 0.06291 | 0.06291 | 0.06022 | 0.0116 | 0.08132 | 0.01302 | 0.04071 | 0.04567 | 0.03074 | 0.10677 | 0.01696 | 0.10046 | 0.00736 | 0.0028 | 0.00385 | 0.00455 | 0.00772 | 0.01732 | 0.00455 | 0.0049 |  |  |  |  |  |  |  |  |  |  |  |  |  |  |  |
| Parmotrema_ultralucens | 0.01231 | 0.10812 | 0.13052 | 0.06876 | 0.02927 | 0.02926 | 0.03072 | 0.03108 | 0.02961 | 0.02999 | 0.02927 | 0.0289 | 0.02963 | 0.02928 | 0.03034 | 0.08195 | 0.16287 | 0.1297 | 0.06872 | 0.06321 | 0.06319 | 0.06248 | 0.06288 | 0.06288 | 0.06094 | 0.01301 | 0.08162 | 0.01372 | 0.04179 | 0.04748 | 0.03145 | 0.10823 | 0.01695 | 0.10036 | 0.00665 | 0.0035 | 0.0042 | 0.00175 | 0.00843 | 0.01945 | 0.00314 | 0.0049 | 0.0035 |  |  |  |  |  |  |  |  |  |  |  |  |  |  |
| Pertusaria_obruta | 0.07261 | 0.11611 | 0.13502 | 0.10171 | 0.07481 | 0.07287 | 0.07723 | 0.07289 | 0.07558 | 0.07763 | 0.0733 | 0.07562 | 0.07563 | 0.07371 | 0.07367 | 0.09523 | 0.1788 | 0.15413 | 0.1062 | 0.09786 | 0.09874 | 0.09992 | 0.1012 | 0.1012 | 0.09872 | 0.07721 | 0.10015 | 0.07717 | 0.08007 | 0.08798 | 0.07371 | 0.10892 | 0.07918 | 0.12577 | 0.07634 | 0.07561 | 0.07638 | 0.07635 | 0.08039 | 0.0819 | 0.07633 | 0.07684 | 0.0729 | 0.0752 |  |  |  |  |  |  |  |  |  |  |  |  |  |
| Pertusaria_ostiolata | 0.06273 | 0.10965 | 0.12942 | 0.09386 | 0.06838 | 0.06724 | 0.07038 | 0.06687 | 0.06838 | 0.07116 | 0.06652 | 0.06917 | 0.06918 | 0.06692 | 0.06725 | 0.08752 | 0.16827 | 0.13997 | 0.09783 | 0.09196 | 0.09044 | 0.09242 | 0.09247 | 0.09247 | 0.09157 | 0.06691 | 0.08899 | 0.06536 | 0.07163 | 0.07447 | 0.06767 | 0.10918 | 0.06618 | 0.11848 | 0.06567 | 0.0657 | 0.06566 | 0.06644 | 0.07039 | 0.0723 | 0.06641 | 0.06615 | 0.06303 | 0.0653 | 0.03883 |  |  |  |  |  |  |  |  |  |  |  |  |
| Pertusaria_plittiana | 0.06123 | 0.11204 | 0.13042 | 0.08631 | 0.06495 | 0.06304 | 0.06733 | 0.06307 | 0.06571 | 0.0681 | 0.06348 | 0.06649 | 0.06575 | 0.06425 | 0.06421 | 0.08699 | 0.1674 | 0.13973 | 0.09207 | 0.08476 | 0.08482 | 0.08409 | 0.08645 | 0.08645 | 0.08479 | 0.0658 | 0.09567 | 0.06388 | 0.07119 | 0.07369 | 0.06388 | 0.10998 | 0.06694 | 0.11886 | 0.06189 | 0.06307 | 0.06303 | 0.0638 | 0.06736 | 0.06964 | 0.06378 | 0.06501 | 0.06115 | 0.06341 | 0.04064 | 0.03103 |  |  |  |  |  |  |  |  |  |  |  |
| Pertusaria_propinqua | 0.0586 | 0.10647 | 0.12309 | 0.08877 | 0.06538 | 0.06425 | 0.06738 | 0.06313 | 0.06614 | 0.06815 | 0.06278 | 0.06617 | 0.06618 | 0.06355 | 0.06351 | 0.08196 | 0.17114 | 0.13641 | 0.09142 | 0.08331 | 0.08333 | 0.08376 | 0.08535 | 0.08535 | 0.0837 | 0.06276 | 0.09109 | 0.06272 | 0.07128 | 0.07222 | 0.06543 | 0.10193 | 0.06315 | 0.11609 | 0.06191 | 0.06119 | 0.06115 | 0.06192 | 0.06388 | 0.06811 | 0.0619 | 0.06313 | 0.05928 | 0.06154 | 0.03959 | 0.02556 | 0.02487 |  |  |  |  |  |  |  |  |  |  |
| Phlyctis_boliviensis | 0.08962 | 0.13385 | 0.15927 | 0.1108 | 0.08816 | 0.08661 | 0.09025 | 0.08776 | 0.08894 | 0.0902 | 0.08704 | 0.08898 | 0.08899 | 0.08707 | 0.08662 | 0.12334 | 0.18032 | 0.13407 | 0.11048 | 0.10263 | 0.10138 | 0.10255 | 0.10468 | 0.10468 | 0.10135 | 0.09235 | 0.11351 | 0.09183 | 0.09658 | 0.10346 | 0.09419 | 0.13517 | 0.09725 | 0.13697 | 0.08994 | 0.09121 | 0.09238 | 0.0888 | 0.09149 | 0.09378 | 0.0907 | 0.09202 | 0.09037 | 0.08955 | 0.10988 | 0.10076 | 0.0988 | 0.09314 |  |  |  |  |  |  |  |  |  |
| Phyllopsora_corallina | 0.05625 | 0.10935 | 0.13273 | 0.06769 | 0.05003 | 0.04962 | 0.05345 | 0.04891 | 0.05037 | 0.05194 | 0.04928 | 0.05113 | 0.0504 | 0.04929 | 0.05037 | 0.08722 | 0.17128 | 0.13387 | 0.08362 | 0.07496 | 0.07525 | 0.07408 | 0.0765 | 0.0765 | 0.07525 | 0.05573 | 0.0947 | 0.0566 | 0.05985 | 0.0648 | 0.04971 | 0.11352 | 0.05506 | 0.11168 | 0.05724 | 0.05504 | 0.05505 | 0.055 | 0.05928 | 0.0645 | 0.05497 | 0.05616 | 0.0539 | 0.05387 | 0.08666 | 0.07799 | 0.07603 | 0.0742 | 0.10208 |  |  |  |  |  |  |  |  |
| Usnea_ceratina | 0.01231 | 0.11032 | 0.13204 | 0.0727 | 0.02999 | 0.02926 | 0.03144 | 0.03072 | 0.03033 | 0.03071 | 0.03035 | 0.03035 | 0.03035 | 0.03036 | 0.02998 | 0.08557 | 0.16555 | 0.13222 | 0.0702 | 0.0643 | 0.06386 | 0.06278 | 0.06393 | 0.06393 | 0.06236 | 0.01231 | 0.08633 | 0.01159 | 0.04106 | 0.0475 | 0.0333 | 0.10628 | 0.02056 | 0.10171 | 0.01517 | 0.01304 | 0.01375 | 0.01373 | 0.0166 | 0.02671 | 0.01374 | 0.01268 | 0.01126 | 0.01267 | 0.07721 | 0.06689 | 0.06541 | 0.0635 | 0.09217 | 0.05771 |  |  |  |  |  |  |  |
| Usnea_cornuta | 0.01408 | 0.11235 | 0.13331 | 0.07545 | 0.03181 | 0.03109 | 0.03327 | 0.03328 | 0.03216 | 0.03253 | 0.03292 | 0.03217 | 0.03217 | 0.03293 | 0.03254 | 0.08831 | 0.16562 | 0.13186 | 0.0706 | 0.0655 | 0.06428 | 0.0632 | 0.06513 | 0.06513 | 0.06278 | 0.01551 | 0.08478 | 0.01408 | 0.04217 | 0.04936 | 0.0366 | 0.11071 | 0.02237 | 0.10531 | 0.01838 | 0.01552 | 0.01552 | 0.01587 | 0.01838 | 0.02998 | 0.01623 | 0.01553 | 0.01446 | 0.0141 | 0.07762 | 0.06729 | 0.06656 | 0.0654 | 0.09298 | 0.06001 | 0.00385 |  |  |  |  |  |  |
| Usnea_halei | 0.01444 | 0.11236 | 0.13292 | 0.07462 | 0.03362 | 0.03361 | 0.03583 | 0.03436 | 0.03397 | 0.03509 | 0.03254 | 0.03326 | 0.03399 | 0.03328 | 0.03362 | 0.08839 | 0.16686 | 0.13141 | 0.07404 | 0.06698 | 0.06616 | 0.06508 | 0.06698 | 0.06698 | 0.06616 | 0.01658 | 0.08477 | 0.01514 | 0.04475 | 0.04862 | 0.03771 | 0.11114 | 0.02201 | 0.10572 | 0.01873 | 0.01589 | 0.0166 | 0.01659 | 0.01875 | 0.03035 | 0.01516 | 0.01624 | 0.01482 | 0.01552 | 0.07873 | 0.0684 | 0.06579 | 0.06387 | 0.09371 | 0.05887 | 0.0056 | 0.00595 |  |  |  |  |  |
| Usnea_mutibilis | 0.01551 | 0.1104 | 0.13299 | 0.07593 | 0.03259 | 0.03258 | 0.03294 | 0.03369 | 0.03293 | 0.0322 | 0.03368 | 0.03295 | 0.03295 | 0.03369 | 0.03295 | 0.0861 | 0.16891 | 0.13488 | 0.07292 | 0.06817 | 0.0666 | 0.06627 | 0.06705 | 0.06705 | 0.0651 | 0.0166 | 0.08804 | 0.01552 | 0.04299 | 0.05019 | 0.03626 | 0.10803 | 0.0231 | 0.10299 | 0.0191 | 0.01624 | 0.0166 | 0.01694 | 0.01911 | 0.03071 | 0.01695 | 0.01589 | 0.01481 | 0.01516 | 0.07965 | 0.06813 | 0.06701 | 0.06473 | 0.09349 | 0.05969 | 0.00666 | 0.00878 | 0.01089 |  |  |  |  |
| Usnea_pensylvanica | 0.01266 | 0.11031 | 0.13158 | 0.07232 | 0.03035 | 0.02961 | 0.03252 | 0.03108 | 0.03069 | 0.03179 | 0.03071 | 0.0307 | 0.03071 | 0.03072 | 0.03034 | 0.08594 | 0.16786 | 0.1328 | 0.07105 | 0.06514 | 0.0647 | 0.06362 | 0.06477 | 0.06477 | 0.0632 | 0.01337 | 0.08719 | 0.01267 | 0.04177 | 0.04786 | 0.03366 | 0.10744 | 0.02092 | 0.10209 | 0.01623 | 0.0141 | 0.0141 | 0.01479 | 0.0184 | 0.02778 | 0.01409 | 0.01374 | 0.01232 | 0.01302 | 0.07834 | 0.06721 | 0.06653 | 0.06382 | 0.09423 | 0.0577 | 0.00385 | 0.00631 | 0.00807 | 0.00843 |  |  |  |
| Usnea_subfusca | 0.01159 | 0.10707 | 0.12869 | 0.07037 | 0.02853 | 0.02852 | 0.0307 | 0.02998 | 0.02887 | 0.02996 | 0.02961 | 0.02888 | 0.02889 | 0.0289 | 0.02852 | 0.08243 | 0.16864 | 0.13191 | 0.06987 | 0.06397 | 0.06278 | 0.06245 | 0.06285 | 0.06285 | 0.06203 | 0.01231 | 0.08599 | 0.01231 | 0.03994 | 0.04525 | 0.03111 | 0.10463 | 0.01841 | 0.09968 | 0.01516 | 0.01303 | 0.01232 | 0.01372 | 0.01661 | 0.02669 | 0.01302 | 0.01267 | 0.01125 | 0.01195 | 0.07753 | 0.06647 | 0.06499 | 0.06308 | 0.09301 | 0.05544 | 0.00349 | 0.00595 | 0.00771 | 0.00737 | 0.00384 |  |  |
| Usnea_subgracilis | 0.01196 | 0.10995 | 0.13167 | 0.07312 | 0.03109 | 0.03035 | 0.03327 | 0.0311 | 0.03143 | 0.03253 | 0.03073 | 0.03144 | 0.03145 | 0.03074 | 0.03108 | 0.08599 | 0.16686 | 0.13138 | 0.07061 | 0.06471 | 0.06427 | 0.06319 | 0.06434 | 0.06434 | 0.06277 | 0.01409 | 0.08554 | 0.01195 | 0.04291 | 0.04638 | 0.0344 | 0.1075 | 0.02022 | 0.10373 | 0.01624 | 0.0141 | 0.01411 | 0.0148 | 0.01696 | 0.0278 | 0.0141 | 0.01375 | 0.01233 | 0.01303 | 0.07757 | 0.0665 | 0.06502 | 0.06311 | 0.09176 | 0.05773 | 0.00244 | 0.00279 | 0.00385 | 0.00772 | 0.0042 | 0.00385 |  |
| Usnea_subscabrosa | 0.01372 | 0.10946 | 0.1307 | 0.0719 | 0.03144 | 0.0307 | 0.03289 | 0.03145 | 0.03178 | 0.03215 | 0.03108 | 0.03179 | 0.0318 | 0.03109 | 0.03143 | 0.08475 | 0.16735 | 0.13435 | 0.07258 | 0.06666 | 0.06622 | 0.06514 | 0.06629 | 0.06629 | 0.06471 | 0.01373 | 0.08677 | 0.01373 | 0.04252 | 0.04747 | 0.03475 | 0.10498 | 0.01984 | 0.1033 | 0.01659 | 0.01445 | 0.01446 | 0.01515 | 0.01876 | 0.02815 | 0.01445 | 0.01409 | 0.01267 | 0.01338 | 0.07716 | 0.0661 | 0.06462 | 0.06271 | 0.09464 | 0.0573 | 0.0028 | 0.00596 | 0.00701 | 0.00808 | 0.00455 | 0.00419 | 0.00385 |
